# Importance of Site Diversity and Connectivity in Electrochemical CO Reduction on Cu

**DOI:** 10.1021/acscatal.3c05904

**Published:** 2024-02-14

**Authors:** Chansol Kim, Nitish Govindarajan, Sydney Hemenway, Junho Park, Anya Zoraster, Calton J. Kong, Rajiv Ramanujam Prabhakar, Joel B. Varley, Hee-Tae Jung, Christopher Hahn, Joel W. Ager

**Affiliations:** † Chemical Sciences Division, Lawrence Berkeley National Laboratory, Berkeley, California 94720, United States; ‡ Department of Chemical and Biomolecular Engineering, Korea Advanced Institute of Science and Technology (KAIST), 291 Daehak-ro, Yuseong-gu, Daejeon 34141, South Korea; § Materials Science Division, Lawrence Livermore National Laboratory, Livermore, California 94550, United States; ∥ Department of Materials Science and Engineering, University of California, Berkeley, Berkeley, California 94720, United States; ⊥ Department of Chemical and Biochemical Engineering, University of California, Berkeley, Berkeley, California 94720, United States; # Materials Sciences Division, Lawrence Berkeley National Laboratory, Berkeley, California 94720, United States; ¶ Clean Energy Research Center, Korea Institute of Science and Technology (KIST), Seoul 02792, South Korea

**Keywords:** electrocatalysis, chemical transient kinetics, CO reduction, microkinetic modeling, catalytic mechanism

## Abstract

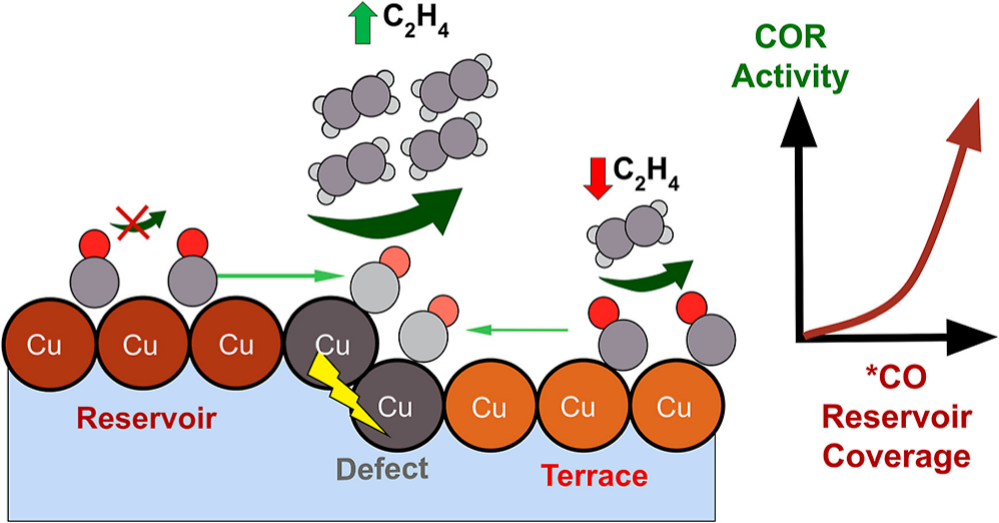

Electrochemical CO_2_ reduction on Cu is a promising approach to produce value-added chemicals using renewable feedstocks, yet various Cu preparations have led to differences in activity and selectivity toward single and multicarbon products. Here, we find, surprisingly, that the effective catalytic activity toward ethylene improves when there is a larger fraction of less active sites acting as reservoirs of *CO on the surface of Cu nanoparticle electrocatalysts. In an adaptation of chemical transient kinetics to electrocatalysis, we measure the dynamic response of a gas diffusion electrode (GDE) cell when the feed gas is abruptly switched between Ar (inert) and CO. When switching from Ar to CO, CO reduction (COR) begins promptly, but when switching from CO to Ar, COR can be maintained for several seconds (delay time) despite the absence of the CO reactant in the gas phase. A three-site microkinetic model captures the observed dynamic behavior and shows that Cu catalysts exhibiting delay times have a less active *CO reservoir that exhibits fast diffusion to active sites. The observed delay times and the estimated *CO reservoir sizes are affected by catalyst preparation, applied potential, and microenvironment (electrolyte cation identity, electrolyte pH, and CO partial pressure). Notably, we estimate that the *CO reservoir surface coverage can be as high as 88 ± 7% on oxide-derived Cu (OD-Cu) at high overpotentials (−1.52 V *vs* SHE) and this increases in reservoir coverage coincide with increased turnover frequencies to ethylene. We also estimate that *CO can travel substantial distances (up to 10s of nm) prior to desorption or reaction. It appears that active C–C coupling sites by themselves do not control selectivity to C_2+_ products in electrochemical COR; the supply of CO to those sites is also a crucial factor. More generally, the overall activity of Cu electrocatalysts cannot be approximated from linear combinations of individual site activities. Future designs must consider the diversity of the catalyst network and account for intersite transportation pathways.

## Introduction

There is intense interest in the role of Cu as an electrocatalyst in electrochemical CO_2_ reduction (EC-CO_2_R) because potentially valuable products such as ethylene and ethanol can be made at industrially viable current densities.^1,2^ Among metal electrocatalysts, Cu is unique in its ability to selectively produce C–C coupled products, although exclusive selectivity to a specific product is yet to be achieved.^3^ It has been known for many years that EC-CO_2_R on Cu proceeds through an adsorbed CO intermediate (*CO) and that its hydrogenated and dimerized forms are involved in the rate-determining step for C_1_ and C_2+_ product formation, respectively.^4,5^ Accordingly, many experimental and theory efforts have focused on evaluating the CO binding energy on various Cu surfaces and on determining the identity of the active sites responsible for C–C coupling.^6−10^ At the same time, there is increasing attention of the role of the active site microenvironment, including effects associated with the electrolyte including pH, electric fields, and specific cations.^11−18^

Still, there is evidence that focusing solely on the Cu active sites may not yield a complete picture of activity and selectivity in EC-CO_2_R, particularly for nanostructured forms used in high-current density gas diffusion electrode (GDE) cells. The first consideration is the wide range of CO binding energies that is predicted for Cu nanoparticles,^19^ combined with experimental evidence that *CO is mobile under reaction conditions.^20^ There is also experimental evidence linking the distribution of the *CO binding energies to activity. For example, the temperature-programmed desorption (TPD) study of Verdaguer-Casadevall *et al.* found that Cu subjected to the oxidation–reduction cycling (“oxide-derived Cu,” OD-Cu) had both larger fraction of strong CO binding sites and a higher activity for CO_2_R to ethylene in H-cell measurements, suggesting a possible correlation between the two quantities.^6^ Furthermore, isotopic labeling experiments have provided evidence for product-selective sites on oxide-derived Cu and similar high surface area Cu electrocatalysts.^21,22^ Taken as a whole, those reports suggest that a full understanding of Cu CO_2_R electrocatalysts will necessitate looking beyond individual active sites and their nearby microenvironments.

To this end, significant efforts have been made to understand the relationship between various active sites and intermediates and the resultant product distribution in EC-CO_2_R on Cu. Several studies have performed *in situ* spectroscopy (surface-enhanced Raman and infrared absorption) during EC-CO_2_R on both low-index and nanostructured Cu surfaces.^20,23−26^ Also, there has been substantial progress using *ab initio* calculations of intermediate binding energies and barriers to elucidate the mechanism and, ambitiously, to suggest methods to improve selectivity.^5,9,16,27−32^ Still, CO_2_ reduction on Cu has been a difficult system to study, particularly in the high current density conditions found in gas diffusion electrode (GDE) environments.^33^ Furthermore, Cu is well known to restructure under EC-CO_2_R conditions.^34−36^ Accordingly, it is still difficult to directly compare the results from experiments performed with nanostructured Cu to predictions of first-principles-based simulations.^19,33,37^

Noting that many prior experimental/theory reports compare steady-state partial current densities and Faradaic efficiencies obtained at a given potential, we posited that additional mechanistic insights could be obtained by monitoring the dynamic behavior of an electrolysis cell. We derived specific motivation from the use of chemical transient kinetics (CTK) and related approaches to discern mechanisms in heterogeneous catalysis.^38−41^ In EC-CO_2_R, mass spectrometric approaches have been used to monitor products as a function of potential, most often in H-cells as low current density.^42−44^ Here, we targeted the high-current density conditions found in GDE cells operated at a fixed potential in order to mitigate against effects due to catalyst restructuring. Also, we focus on CO reduction (COR) because its use avoids all issues associated with CO_2_ reacting with OH^–^ to form bicarbonate (CO_2_ pumping), which occurs in high current density GDEs.^45^

Our experimental protocol is simple: the feed gas to a GDE cell with a Cu cathode is repeatedly switched between Ar and CO at a constant applied potential. With an Ar feed, the Cu electrocatalyst will perform the hydrogen evolution reaction (HER); with a CO feed, the COR and HER will both occur. We found that the transition from HER to COR/HER (and its reverse) could be monitored with excellent time resolution by precisely measuring the mass flow exiting the cell with an appropriately selected mass flow meter (MFM). When the gas feed is switched from Ar to CO, the transition from the HER to COR/HER is rapid. However, surprisingly, when the gas feed is switched from CO to Ar, COR can proceed at the same rate for several seconds (delay time). Control experiments establish that some Cu preparations, most notably oxide-derived Cu (OD-Cu), are able to maintain COR activity in the absence of the gas phase precursor because they have reservoir sites that bind CO but do not convert it. We introduce a three-site microkinetic model that shows that this behavior originates from the interplay of a less active *CO reservoir and the diffusion of CO from that reservoir to more active sites on the Cu surface. Ultimately, the work shows that EC-CO_2_R catalyst design needs a broader scope: the distribution of active sites and less active sites must be optimized to control surface CO availability and C_2+_ activity.

## Results and Discussion

### Chemical Transient Kinetics Measurements of Electrochemical CO Reduction

**Figure 1:**
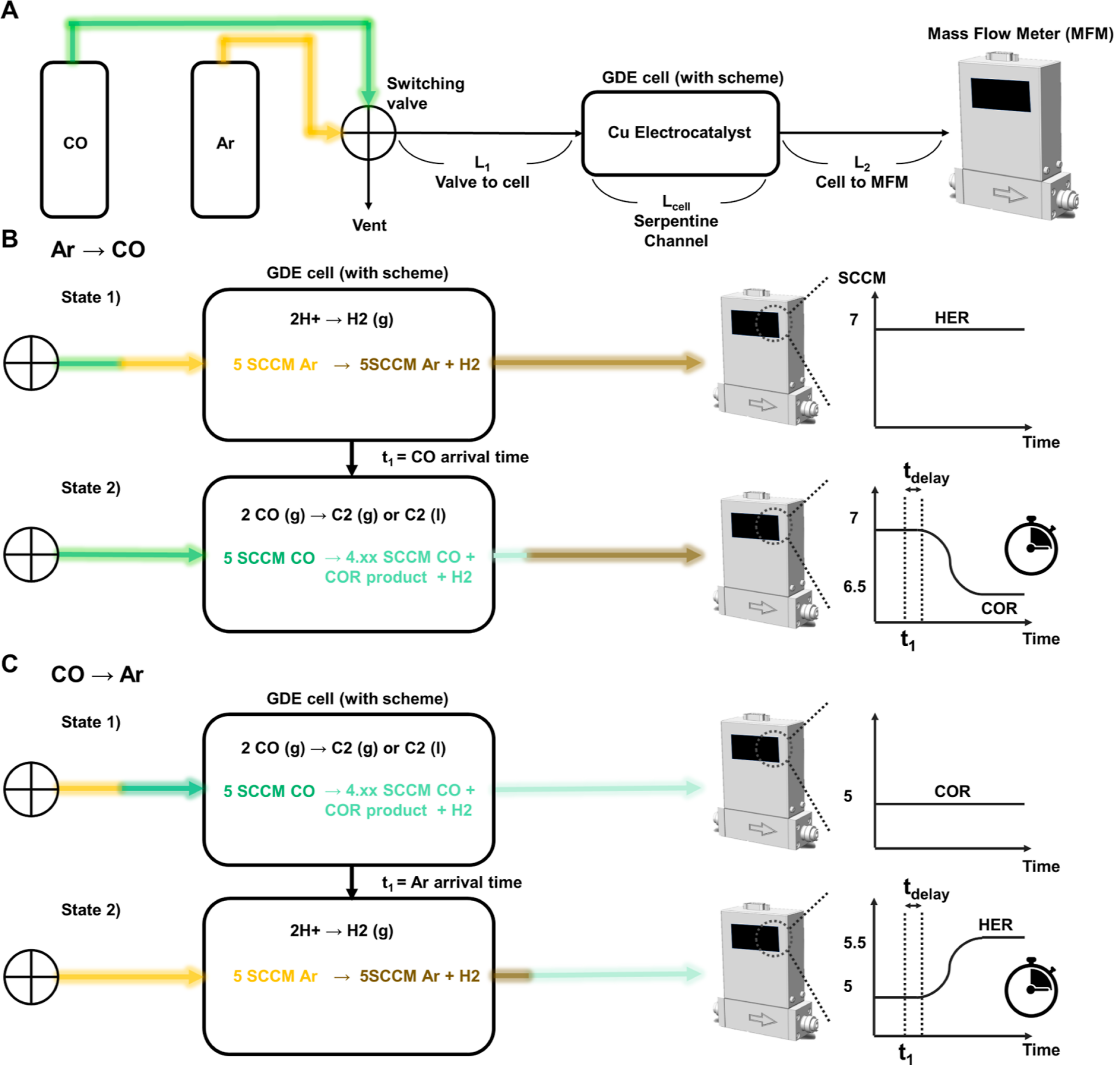
Chemical transient kinetics measurements of electrochemical CO reduction. (A) CO and Ar are supplied at 5 sccm to a switching valve which directs the gas either to the vent or to the inlet of the GDE cell. The mass flow exiting the cell is measured by the MFM calibrated to CO. (B) When Ar is supplied to the cell, the mass flow out of the cell is higher than the supply due to HER, which does not consume the feed gas (state 1). The mass flow reading is also elevated due to the larger viscosity of Ar as compared to that of CO. When the gas in the GDE cell is switched to CO, the mass flow will be reduced due to the consumption of CO by COR to C_2+_ products (state 2). We define *t*_1_ as the time required for the gas to arrive at the entrance of the GDE cell after switching: *t*_1_ = 6.60 s for our standard setup of *L*_1_ = 30.8 cm and mean flow velocity *u̲* = 280 cm/min. Measurement of gas velocity and consideration of effects due to Taylor–Aris dispersion are described in the Supporting Information. (C) When CO is supplied to the cell, the exit mass flow will be lower than the supply (state 1), unless HER is dominant. When the feed is switched to Ar, the mass flow will increase as the HER becomes the dominant reaction (state 2). In both (B,C), time *t*_delay_ accounts for any delay in the change in the products produced by the GDE beyond *t*_1_.

Our experimental setup allows for fast switching of the feed gas supplied to the electrolysis cell, as shown in Figure 1A. We used gas diffusion cells of typical design with Cu on carbon paper functioning as the GDE and a flowing aqueous electrolyte. Most experiments were performed with commercially purchased 25 nm diameter Cu nanoparticles subjected to an oxidation treatment (OD-Cu NPs), with as-purchased (Cu NPs), post annealed (OD Cu NP/post annealed), and sputtered Cu serving as controls. A full description of experimental procedures and the COR Faradaic efficiencies and partial current densities for all catalysts employed in the study is given in the Supporting Information.

We alternated between identical flows (5 sccm) of either Ar or CO and used a downstream MFM to monitor the mass flow exiting the reactor (Figure 1A). Importantly, changes in the MFM reading reflect, essentially instantaneously, changes in the chemistry occurring in the GDE as the gas is changed. For example, as shown in Figure 1B, when Ar is provided to the cell, the mass flow exiting the cell is higher than the supply because HER produces a gas phase product that does not consume Ar (actual MFM reading will depend on the gas composition, see the Supporting Information). Conversely, when the gas flow is switched to CO, the mass flow exiting the cell will be smaller: the partial current density to the HER will decline and some fraction of CO is consumed to form C_2+_ products. When the gas feed is switched back to Ar (Figure 1C), the mass flow exiting the cell will increase again. Crucially, the response of the MFM to changes in the mass flow is very fast, *ca.* 50 ms (Figure S15); the time resolution of the measurement (∼1.5 s) is instead limited by the finite size of the GDE cell and Taylor–Aris dispersion that mixes Ar and CO (see the Supporting Information and Figure S16). The fast MFM response for both COR and HER conditions is highly reproducible, as shown in the repeat potential pulsing experiments summarized in Figure S19. We do note that there will be a change in the reading when the gas composition at the MFM changes; we chose the distance between the cell and the MFM (*l*_2_ in Figure 1A) such that this will not affect the measurements (see Figure S20).

**Figure 2:**
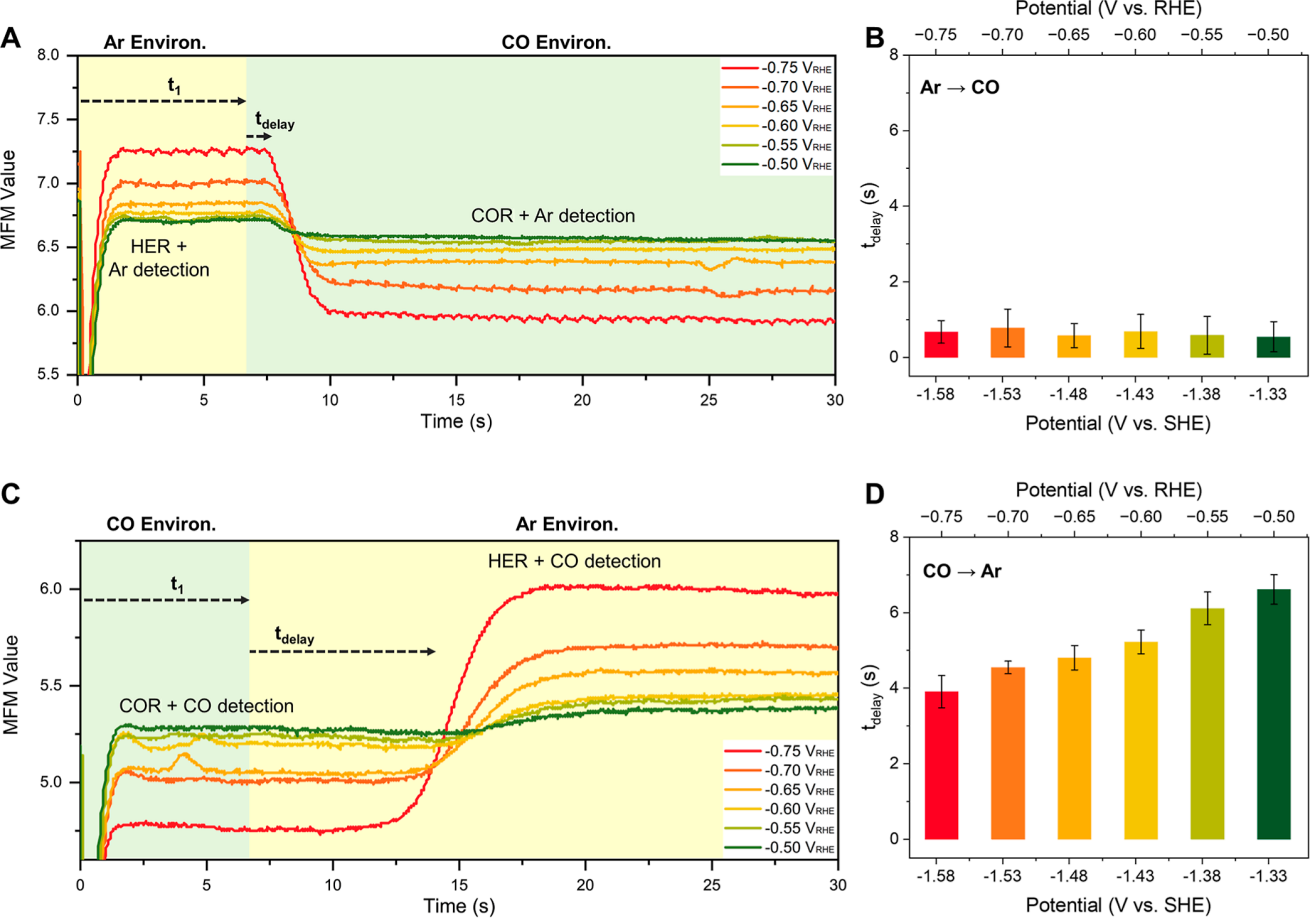
Chemical transient kinetic measurements of COR. (A) MFM reading as gas feed is switched from Ar (yellow shading) to CO (green shading). (B) *t*_delay_ is small and is unchanged as a function of the cathode potential. When the gas feed is switched from CO to Ar (C), HER does not start immediately but instead a delay time of 4–6 s is seen, depending on the cathode potential (D). 25 nm OD-Cu and Nafion on carbon paper, 1 M KOH (pH 14), 1 mL/min electrolyte flow, and 5 sccm Ar/CO flow rate. Error bars are standard deviations from at least 3 repeated measurements.

Due to the relatively weaker binding energies of the intermediates associated with HER on Cu,^37^ we expect that COR would commence very soon after the feed gas is switched from Ar to CO. Figure 2A shows that this is indeed the case for OD-Cu across a range of cathode potentials; the boundary between Ar and CO arrives at the cell entrance at a time *t*_1_ = 6.60 s, and the expected decrease in the mass flow starts essentially immediately (*t*_delay_ ∼ 0.5 s), as shown in Figure 2B. The current in the cell also changes, in excellent correspondence with the mass flow data (Figure S21).

Intriguingly, the kinetics of switching from a CO feed to an Ar feed for an OD-Cu cathode is very different. Referring to Figure 2C, Ar arrives at the GDE at time *t*_1_ but, as clearly evidenced by the unchanging mass flow, COR continues at the same rate for several seconds in spite of the absence of the CO reactant in the gas phase. The delay time decreases from 3.9 to 6.6 s with increasing overpotential, as shown in Figure 2D. These values are larger than our estimated time resolution and thus provide information on the rate at which *CO diffuses on or near the Cu surface to the sites where it eventually reacts and leaves.

### Control Experiments to Discern the Location of the CO Reservoir

Clearly, there is a reservoir of CO somewhere in the system that allows reactant supply to the active sites even when the gas environment of the cell has been changed to inert Ar. Noting that Louisia *et al.*, in CO_2_R measurements performed in an H-cell, had found evidence of a CO reservoir in the near-electrode region,^46^ we performed a series of control experiments to discern the location of the reservoir in our case. Due to the laminar flow (Reynolds number *Re* ∼ 5, see the Supporting Information) employed in this work, we do not expect any holdup of gas in the supply tubing. This unlikely possibility was also ruled out by control experiments showing no significant effect on the delay time when *L*_1_ was varied over more than an order of magnitude (Figure S22). We also considered the possibility of a CO reservoir located in the GDE, but this was ruled out by experiments performed with different thicknesses of the carbon paper (Figure S23). We also calculated the gas transit time through the carbon paper: it is <20 ms, see the Supporting Information, and is too short to explain the much longer delay times. We considered the possibility of physisorption of CO at the Cu and carbon paper interfaces but found that changing the catalyst loading had little effect on the delay time (Figure S25). Also, as we will show later that the size of the reservoir can be >50% of the electrochemically active surface area and is thus too large to be explained this way because the Cu particles only contact the carbon paper with a small part of their surface area. Finally, changing the ionomers used in making the catalyst ink had only a small effect (Figure S24).

In contrast, changing the Cu catalyst had a large effect, as discussed later. These experiments, along with the fact that the delay time depends strongly on potential, show that the CO reservoir is located on the surface of Cu. We note that Gunathunge *et al.*, in a combined spectroscopic and theoretical study, have found evidence of inactive *CO located at bridge sites.^47^ Our results are consistent with this work, although we, as discussed below, do not assign the reservoir to bridge sites only and also do not rule out the possibility of them having non-negligible COR activity.

### Overview of the Microkinetic Model

At minimum, there must be at least two categories of sites on the Cu surface: (1) sites that function as a reservoir by binding CO and (2) active sites that facilitate C–C coupling. Furthermore, there must be a means for CO to travel from the reservoir to the active sites, and the reservoir sites need to be able to bind CO sufficiently strongly to prevent complete desorption during *t*_delay_ but not so strongly as to prevent transport *via* surface diffusion.

Initially, we considered two-site models but found that implausible values of binding energies and diffusion rates were required to predict the observed delay times. However, we find that a three-site microkinetic model can capture the main effects observed experimentally. Although the actual Cu surface under reaction conditions consists of several different site types, our model broadly notionally classifies them into three categories: “reservoir,” “terrace,” and “defects” based on their CO* binding strength, *CO surface diffusivity, and activity toward C–C coupling to form C_2+_ products, as summarized in Table 1; a sensitivity analysis of these values is provided in the Supporting Information Figures S30 and S31.

**Table 1:** Site Types and the Associated Rate Constants Used in the Three-Site Microkinetic Model

site type	activity for C–C coupling	*CO adsorption	*CO diffusion rates
reservoir	low (0.01 s^–1^)	strong binding	to terrace: slow (1 s^–1^)
			to defects: fast (1000 s^–1^)
terrace	moderate (0.1 s^–1^)	moderate binding	to reservoir: fast (100 s^–1^)
			to defects: fast (10 000 s^–1^)
defects	high (1 s^–1^)	strong binding	to reservoir: moderate (10 s^–1^)
			to terrace: slow (0.1 s^–1^)

It is interesting to consider the specific requirements for a reservoir site: it must be relatively inactive toward C–C coupling yet, somewhat paradoxically, support fast *CO diffusion to the defect sites. We surmise that these types of sites could physically correspond to grain boundaries or other types of undercoordinated sites like step edges that have been shown to exhibit strong *CO binding, larger *CO dimerization energetics relative to other facets like Cu(100), high *CO coverages, and fast CO* diffusion along the undercoordinated sites (e.g., along step edges) that can feed interfacial sites.^6,7,12,47−49^

The other two site types that are active toward C–C coupling differ in their *CO binding strengths and relative dimerization rates with the weaker *CO binding exhibiting faster *CO diffusion. We refer to the weaker binding sites as “terraces” and the most active site for C_2_ formation as “defects.” While we again can only speculate the physical identities of these site types in this simplified model, we note that the latter, highest C_2_ activity sites, may correspond to Cu motifs in the vicinity of interfacial regions between structural defects like grain boundaries and ordered facets like Cu(111) and Cu(100), and thus require the presence of reservoir sites. Based on previous calculations and *in situ* studies, the binding energy and activity of the defect sites best corresponds to overcoordinated, square-ensemble sites and “terrace” sites with terrace and atop sites such as Cu(111) and Cu(100) known to be present under CO_2_R and COR conditions.^47^

**Scheme 1:**
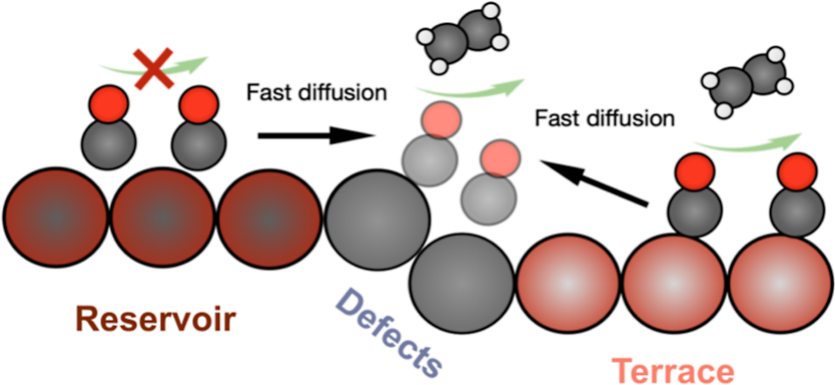
Proposed a Three-Site Model for CO Reduction on Cu The terrace and defect sites are active for C–C coupling, while the reservoir sites are inactive for C–C coupling. The fast diffusion of *CO from the reservoir to the defect sites, together with the presence of a sizeable reservoir *CO coverage, results in the delay time observed in experiments.

The numerical values in Table 1 were chosen as follows. We assign the C–C coupling activity of defect sites to be about an order of magnitude higher than the terrace sites, based on previous observations of small variations in the ECSA-normalized activity of various Cu surface morphologies toward multicarbon products.^3,50^ The reservoir (θ_*CO,r_) and defect sites (θ_*CO,d_) are assumed to be the stronger CO binding sites, while the terrace sites are moderate CO binding (θ_*CO,t_). As a result, there is fast diffusion between the reservoir and defect sites and from the terrace to the reservoir and defect sites. The reservoir sites are close to inactive for C–C coupling, while the terrace sites and the defect sites have moderate to high activity for C–C coupling, respectively. The relationships between the sites are shown in Scheme 1. Further details of the model including justifications for the assumptions and definition of the delay time metric and a sensitivity analysis of the rate constants in Table 1 are provided in the Supporting Information.

### Delay Time Predictions from the Microkinetic Model

**Figure 3:**
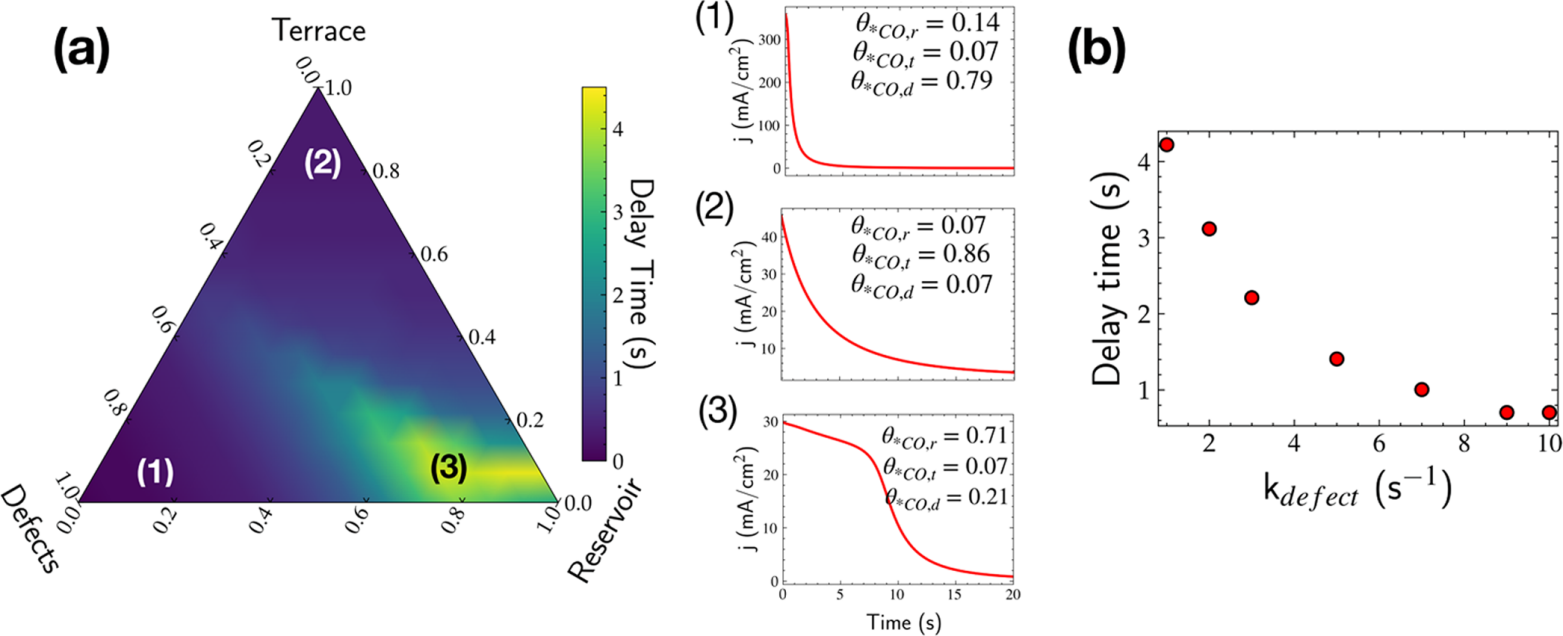
(a) Ternary phase diagram of predicted delay time with *CO coverage on defects, reservoir, and terrace sites (top), and the characteristic COR current density profiles for (1) defect-rich surfaces, (2) terrace-rich, and (3) reservoir-rich Cu surfaces. (b) Dependence of the delay time on the rate constant for C–C coupling on the defect sites. Values used for the *CO coverages on the reservoir (θ_*CO,r_), terrace (θ_*CO,t_), and defects (θ_*CO,d_) to obtain delay time estimations are provided, and rate constants for this figure are consistent with those in Table 1.

Using an initial condition of a CO-saturated surface, the model will predict the time dependence of *CO on each type of site and the overall C–C coupling rate (equivalently, the partial current density to C_2_ products). Using the rates in Table 1, we varied the relative coverages of the three types of sites to find configurations consistent with experimental observations. As shown in Figure 3a, appreciable delay times are predicted intuitively in the corner of the ternary diagram with a large fraction of reservoir sites relative to the other two site types. In terms of our physical model, this could correspond to *CO diffusing along grain boundaries or other extended structural/undercoordinated defects in order to reach other interfacial defects or facets at which C–C coupling becomes significantly more facile. As a result, the system can sustain a given current density for a longer duration, resulting in a current density time profile similar to the experimental observations (Figure 3a (3)).

Naturally, a higher coverage of reservoir *CO can help sustain a given current density for a longer duration, while low reservoir *CO coverages mean that there is not enough supply of *CO from the reservoir to the active sites that would result in a sizable delay time. Here, we note that even though *CO diffusion is faster from the terrace to the defect sites, as the terrace sites are also active for C–C coupling, they are unable to act as “suppliers” in the same way as the reservoir sites that are inactive for C–C coupling. As a result, there is no delay time predicted in the characteristic current density profiles corresponding to the terrace-rich regions of the ternary diagram where terrace *CO is the largest contributor to the simulated current density (Figure 3a (2)). Similarly, our model does not predict any delay time in the defect-rich region (Figure 3a (1)), where only the defect *CO sites contribute to the simulated current density.

Summarizing the insights from the model, an interplay between the *CO coverage on the reservoir and defect sites, with fast surface diffusion from the inactive reservoir sites to the active defect sites, is responsible for the delay time phenomenon. We also find that the delay time reduces with an increase in the rate of C–C coupling on the defect sites (*k*_defect_) due to faster consumption of *CO, resulting in the current density being sustained for a shorter duration. Therefore, we would also expect delay times to reduce with increase in the applied potential, as the rate of C–C coupling that is limited by potential-dependent CO–CO dimerization increases with the potential.^14^

### CO Reservoir on Cu Is Strongly Affected by Catalyst Preparation

**Figure 4:**
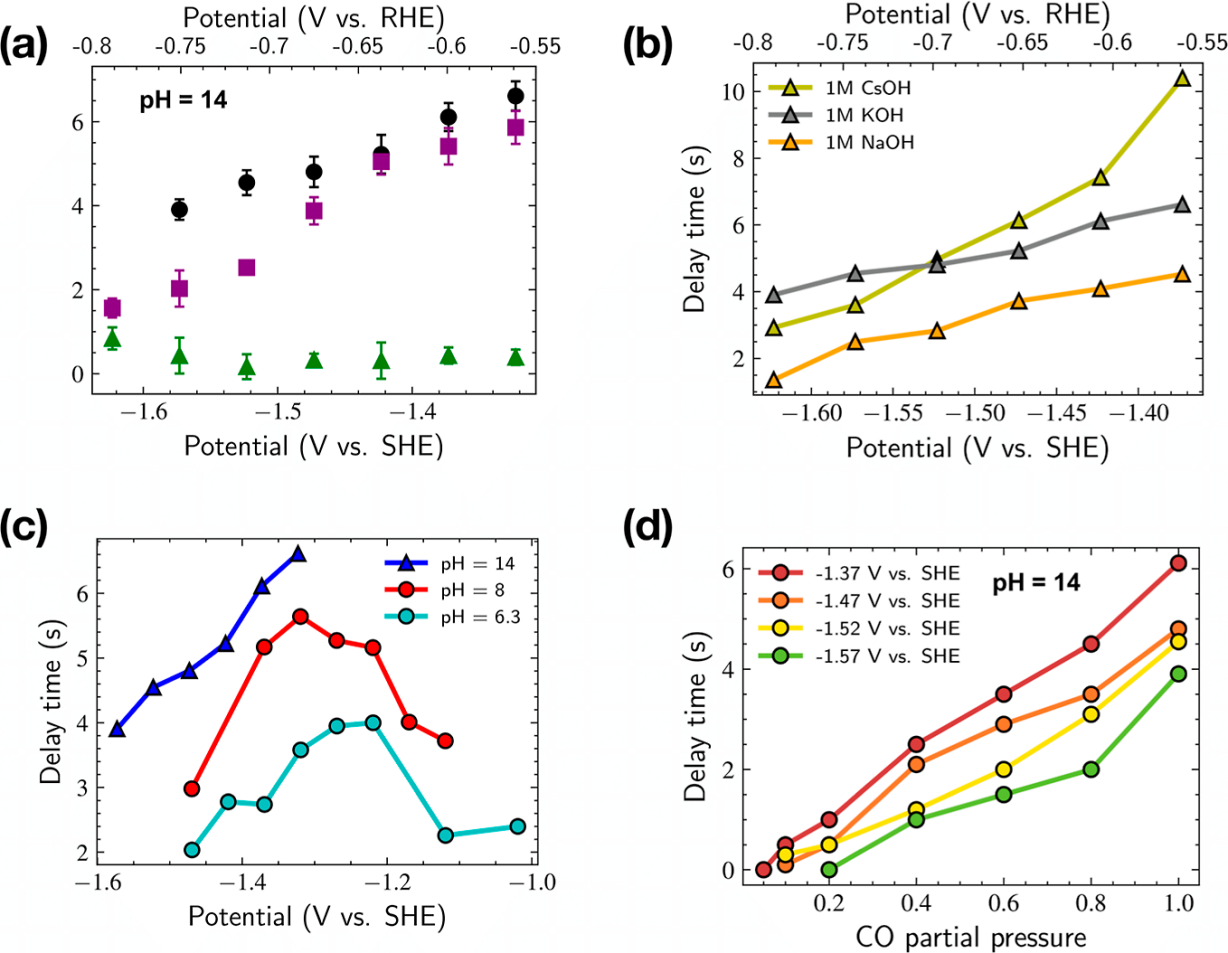
Delay time for different potentials with changes in (a) catalyst preparation, (b) cation identity, (c) electrolyte pH, and (d) CO partial pressure. Unless otherwise noted in the legend, all system conditions are 25 nm OD-Cu and Nafion on carbon paper, 1 M KOH (pH 14), 1 mL/min electrolyte flow, and 5 sccm Ar/CO flow rate.

Experiments presented to this point have used OD-Cu, as this preparation yields the largest geometric current densities for COR and C_2_ products. If the CO reservoir is associated with strong CO binding sites on OD-Cu identified by Verdaguer-Casadevall *et al.*,^6^ we would expect other catalyst preparations to have different, most likely shorter, delay times. Data shown in Figure 4a support this hypothesis. Postannealing OD-Cu NPs reduces the delay time and the as-received Cu NPs have essentially zero delay time. Clearly, the nature of the Cu surface influences the CO reservoir, presumably by sampling the large space of possible CO binding energies, which is likely present in OD-Cu.^19^ Based on the results from the microkinetic model where we find a strong relationship between the reservoir CO coverage and delay times, OD-Cu should also have the highest reservoir site population among the investigated catalyst preparations. We will focus on OD-Cu NPs in the remainder of the manuscript.

### Cation Identity

Previous studies have shown that alkali metal cations with a smaller hydration radius (larger ionic radius) can enhance *CO stabilization and C–C coupling in electrochemical CO_2_ and CO reduction *via* dipole–field interactions between the adsorbate and the cation at the Helmholtz layer.^18,51^ To test our hypothesis that the correlation between *CO binding, C–C coupling, and delay time could be altered by changing the cation identity in the electrolyte, we performed delay time measurements with three different alkali metal cations with decreasing hydration radius (Na^+^, K^+^, and Cs^+^) using the same OD-Cu NPs (Figure 4b). We find the trends in delay time at more negative potentials to correlate well with the hydration radius of the cation, where cations with smaller hydration radius likely increase the concentration of reservoir *CO, leading to longer delay times. This positive correlation between the reservoir size and delay time is self-consistent with Figure 3a. With increasing cathodic potentials, Cs^+^ seems to exhibit a steeper decrease in delay time that might be related to its higher intrinsic rate for C–C coupling compared to Na^+^.^18^

### Electrolyte pH

It is well known that the *CO dimerization step, the putative rate-limiting step for COR to C_2_ products on Cu, is pH independent (*vs* SHE) as it does not involve proton transfer.^14^ Chang *et al.* find the electrolyte pH to influence the surface speciation of Cu, such that substantial differences in the surface speciation and adsorption configuration of CO are observed even at the same SHE potentials.^52^ Additionally, computational studies have observed the binding energy of *CO to depend on the electrolyte pH and applied potential.^12^ This motivated us to measure the delay time as a function of the pH and applied potential (Figure 4c). We find longer delay times for higher pH values at the same SHE potential and posit that the origin of this pH dependence is likely due to the significant surface reconstruction of Cu under alkaline environments.^52,53^ The surface reconstruction results in an increase in the reservoir site density with the electrolyte pH, resulting in the observed increase in delay time for the same SHE potential (Table S1).

### Partial Pressure of CO

Figure 4d shows the change in the delay time with the CO partial pressure of the CO (0.1–1 bar) for a range of potentials. Previous studies have observed sensitivity of C_2_ products to CO partial pressure, where ethylene follows second-order kinetics for CO pressures between 0.5 and 1 bar.^13^ We find that higher CO partial pressures result in larger delay times, most likely due to the increase in the reservoir *CO coverage with increased CO partial pressure. Similar to the previous observations, the delay time decreases at more negative potentials at a given CO partial pressure.

In all of the above scenarios, we find that the delay time is shortened at more negative potentials, which is consistent with the microkinetic model, where overpotential-induced higher C–C coupling rates of the active sites lead to a reduction in delay time (Figure 3b). In summary, our observations indicate that the delay time depends on several factors including the applied potential, the catalyst preparation, and the reaction microenvironment including the cation identity, electrolyte pH, and the CO partial pressure.

### Role of Reservoir Sites on Activity for Ethylene Production

Confident in our hypothesis that the reservoir *CO is adsorbed to stronger binding sites on the Cu surface, we now calculate its size. Specifically, the *CO reservoir size denotes the total amount of *CO needed to maintain the COR rate to ethylene for a given delay time in a 1 cm^2^ geometric area and corresponds to the *CO supplied from a stronger binding (reservoir) site to the defect sites in our model. First, we calculate the amount of CO needed to maintain COR during the delay period, using the partial current densities obtained from FE measurements under steady-state conditions. This yields reservoir sizes as high as 5.90 × 10^–7^ mol cm^–2^ (25 nm OD-Cu, 1 M KOH at −1.573 V *vs* SHE). This value is divided by our estimate of the total number of Cu sites that we obtained from the electrochemical active surface area (ECSA) analysis (Table S1). In agreement with our physical interpretation, reservoir coverage is always less than 100% of the surface, a maximum value of 88 ± 7% (25 nm OD-Cu, 1 M KOH at −1.523 V *vs* SHE).

We add nuance to this picture by calculating the steady-state-combined TOF to ethylene for the active sites (assumed here to be all sites not in the reservoir: the sum of defect and terrace sites). The largest values we obtain, 1–2 s^–1^, are in line with some recent measurements.^54^ At a single potential, there is a clear trend of increasing combined TOF to ethylene with increasing reservoir size (Figure S29a). This trend is also found when all data in study are compared, as shown in Figure S29b, although pH is also a significant factor, with the highest TOFs being observed at pH 14.

**Figure 5:**
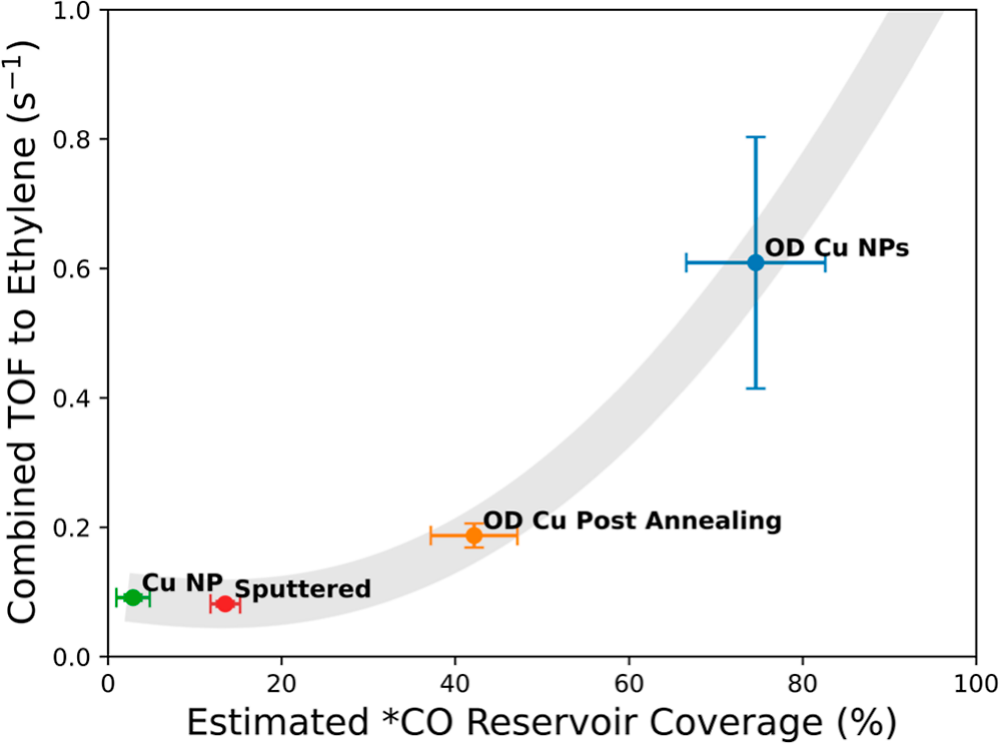
Correlation between the estimated reservoir *CO coverage and the combined TOF to ethylene. System conditions are various Cu preparations on carbon paper tested at −1.473 V *vs* SHE, 1 M KOH (pH 14), 1 mL/min electrolyte, and 5 sccm CO/Ar gas flow rate. Error bars are based on at least 3 measurements of delay time and of partial current densities obtained from gas chromatography during steady-state measurements.

It is interesting to look more deeply into the relationship between reservoir *CO coverage and the combined TOF for the different Cu preparations in the study, as shown in Figure 5, for measurements performed at pH 14 at −1.473 V *vs* SHE. Clearly, the combined TOF increases as a function of reservoir coverage. We interpret this to mean that with the same driving force (intrinsic activity of an active site), the overall TOF is controlled by the reservoir population. Somewhat counterintuitively, when active sites compose a smaller portion of the surface, they are more productive (i.e., have higher combined TOF). This suggests that CO supply on the surface (from the reservoir to the active site) can improve the “effective” activity of the active site. This understanding is along the lines of previous work from Mangione *et al.*, where dual-facet synergy between {100} and {110} interfaces improves C–C coupling rates.^55^

### Broader Scope Is Needed for the Design of Selective COR Electrocatalysts

Due to the interplay between the reservoir and active sites, the overall catalytic activity of the surface cannot be approximated from linear combinations of individual site activities. Jørgensen and Grönbeck, in a kinetic Monte Carlo (KMC) study of thermal CO oxidation on Pt nanoparticles, reached a similar conclusion.^56^ For these reasons, future studies may need to consider larger communities of sites or approximate an “effective site” composed of multiple atomic locations.^57^

An alternative hypothesis is that the presence of reservoir sites coincides with a higher defect site fraction within the combined active sites, as possibly suggested by the interpretation that our defect-type sites may correspond to interfacial binding motifs at the boundary of reservoir and terrace-type sites. Although we currently do not have evidence for such a hypothesis, it could be consistent with the correlations between the density of grain boundaries and CO_2_R and COR activity reported by Kanan and co-workers.^58^

### Role of CO Diffusion

We further estimated the maximal surface diffusion distance of CO on Cu to understand the interaction distance that intermediates might explore during a delay time. We estimate that prior to reaction and/or desorption, CO can travel over distances of *ca.* 10 nm, the same order of magnitude as the diameter of the nanoparticles used in this work. During this time, the *CO will encounter ∼10–100 active sites prior to the reaction (assuming 80% reservoir coverage, as observed at −1.573 V with the preparation of 25 nm OD-Cu dispersed with Nafion on C paper at 1 M KOH and 1 bar CO). Clearly, this finding highlights the need to move beyond models involving stationary adsorbates. During longer diffusion distances and correspondingly longer residence times, intermediates may reorient, influence local pH, modify local electric fields, or otherwise change microenvironment and activity.^59^ Therefore, possible diffusion mechanisms and modes (aqueous, surface, and porous layers) must be considered in the design of CO_2_R and COR catalyst systems, which will be selective for multicarbon products.

### Limitations of the Study and Future Scope

While the three-site microkinetic model provides a good explanation of the experimentally observed delay times for the catalysts investigated in this study, the precise identity of the “reservoir,” “terrace,” and “defect” sites is unknown. It may be possible to identify them *via* time-resolved spectroscopy under conditions where their relative populations are changing.^60^ Still, some limitations of these types of studies should be recognized. Raman spectroscopy can detect *CO but is not quantitative due to the strong surface enhancement effect on Cu. Surface-sensitive infrared techniques will be challenging to employ in the environment of our GDE cell (opaque carbon paper and IR-absorbing water). Also, the strength of absorption features due to *CO can be nonlinear in coverage due to dynamical dipole coupling.^61^ Time-resolved product measurements performed under dynamic conditions should be insightful as well.^44,62,63^

Furthermore, our microkinetic model is zero-dimensional, with the spatial relationships between the different types being captured in an effective diffusion coefficient. Clearly, the present work highlights the need to go beyond mean-field microkinetic models that are currently the state-of-the-art in studying electrocatalytic reaction mechanisms.^64^ Future work in spatially resolved KMC-type approaches that can account for multiple site types and their interaction, accurate site statistics, intersite diffusion, and explicit adsorbate–adsorbate interactions will be important in order to capture the complex interplay between multiple site types on the Cu surface, *CO coverages, and their effects on the underlying reaction mechanism, catalytic activity, and product selectivity.^65,66^

At the system level, careful measurement of the mass flow could be used as a real-time measurement of the overall product distribution. This method could also be used to investigate the origin of phenomena such as electrode flooding and the periodic CO_2_R/HER oscillations which have been observed in membrane electrode assembly (MEA)-type cells.^67,68^

Finally, we studied only a single-component catalyst. It is plausible that, as a result of the insights regarding the importance of reservoir sites, it may be possible to improve the design of the tandem CO_2_R catalyst system, most of which rely on a CO intermediate.^69−72^ In this context, our observations suggest that the design of high-performance electrocatalyst systems should optimize not only for the activity and selectivity of the active sites but also for the supply of intermediates to them.

## Conclusions and Outlook

We used chemical transient kinetic analysis to understand the diversity of catalytically relevant sites on Cu during electrochemical COR in a GDE flow cell. The gas feed to the cell is switched abruptly between Ar and CO; precise measurement of the gas flow exiting the cell is used for the real-time assessment of overall product formation rates (HER *vs* COR to C_2+_ products). COR begins immediately when the gas is switched from Ar to CO, while COR can proceed for several seconds (delay time) when the gas feed is switched back to Ar. The delay time depends on the catalyst preparation, being the longest on OD-Cu. A series of control experiments show that the delay time effect is due the existence of a reservoir of CO on the Cu surface.

A three-site microkinetic model captures the experimental observations very well, including the effects of electrode potential, electrolyte cation, and CO partial pressure. The model assumes three general categories of sites: reservoir sites with strong CO binding but low activity for C–C coupling, terrace sites with moderate CO binding and C–C coupling activity, and defect sites with strong CO binding and high C–C coupling activity. Catalysts that are active for C_2+_ products, such as OD-Cu, counterintuitively, have the highest fraction of less-active reservoir sites. However, the fast diffusion of *CO from the reservoir to the defect site leads to an overall higher turnover frequency compared to catalysts with fewer reservoir sites.

We estimate that for OD-Cu, *CO can diffuse distances on the order of the diameter of the catalyst nanoparticles. Additionally, we observe that the overall catalytic activity cannot be approximated from linear combinations of individual site activities. These findings emphasize that designs for active and selective CO_2_R and COR catalyst systems must consider the diverse catalyst network and intersite transportation pathways.

## Data Availability

The data that supports the findings of this study are openly available on Zenodo at https://zenodo.org/doi/10.5281/zenodo.10109654.

## Supporting Information Available

Structural and electrochemical characterization and analytical chemistry, gas feed switching, measurement of mass flow, time resolution, experimental measurement of delay time and transition time, turnover frequency calculation, microkinetic model, diffusion distance calculation, supplemental tables, supplemental figures, and supplemental references (PDF)
