# Supplementary material for: Importance of Site Diversity and Connectivity in Electrochemical CO Reduction on Cu
Source: ACS Catal. 2024 Feb 14;14(5):3128–38. doi: 10.1021/acscatal.3c05904 (PMC10913037; doi:10.1021/acscatal.3c05904)
Supplement: Supplementary file 1 — cs3c05904_si_001.pdf [file cs3c05904_si_001.pdf]

Supporting Information for

Importance of Site Diversity and Connectivity in Electrochemical CO Reduction on Cu

Chansol Kim[#,abg] Nitish Govindarajan[#,c] Sydney Hemenway,[ad] Junho Park,[ad] Anya Zoraster,[ae] Calton J. Kong,[ad] Rajiv Ramanujam Prabhakar,[a] Joel B. Varley,[c] Hee-Tae Jung,*[b] Christopher Hahn,*[c] and Joel W. Ager*[adf]

[a]Chemical Sciences Division, Lawrence Berkeley National Laboratory, Berkeley, California 94720, United States

[b]Department of Chemical and Biomolecular Engineering, Korea Advanced Institute of Science and Technology (KAIST), 291 Daehak-ro, Yuseong-gu, Daejeon 34141, South Korea

[c]Materials Science Division, Lawrence Livermore National Laboratory, Livermore, California 94550, United States

[d]Department of Materials Science and Engineering, University of California, Berkeley, Berkeley, CA 94720, United States

[e]Department of Chemical and Biochemical Engineering, University of California, Berkeley, Berkeley, CA 94720, United States

[f]Materials Sciences Division, Lawrence Berkeley National Laboratory, Berkeley, California 94720, United States

[g]Clean Energy Research Center, Korea Institute of Science and Technology (KIST), Seoul 02792, South Korea

[#]Equal contribution.

*Corresponding authors. heetae@kaist.ac.kr, hahn31@llnl.gov, jwager@lbl.gov

Contents

Electrocatalytic materials and methods 3

Structural and electrochemical characterization and analytical chemistry 6

Gas feed switching 9

Measurement of mass flow and gas feed switching 9

Time resolution 11

Experimental measurement of delay time 15

Reservoir Size and Turnover Frequency Calculation 17

Microkinetic model 18

Diffusion Distances Calculation 20

Supplemental tables 22

Supplemental figures 25

Supplemental references 51

Electrocatalytic materials and methods

Materials

All chemical reagents were used as purchased without further purification. As-purchased active materials include 25 nm TEM Cu nanoparticles (Sigma-Aldrich), 40-60 nm Cu-NPs (Sigma-Aldrich), and 5 µm Cu particles (Sigma-Aldrich). Dispersions were created using isopropyl alcohol (99.5% VWR Chemicals). The following ionomers were used throughout experiments: Nafion (Ion Power, Chemours D521), Sustainion (Dioxide Materials, XA-9 in ethanol), Fumion (FuelCellStore FAA-3-SOLUT-10). Catalysts were supported by 315 µm carbon paper (Sigracet 39BB, FuelCellStore), 420 µm carbon paper (Sigracet 10 BB, FuelCellStore), and 215 µm carbon paper (Sigracet 22BB, FuelCellStore).

Electrolyte chemicals used include potassium hydroxide (85%, Sigma-Aldrich), cesium hydroxide (99.5%, Sigma-Aldrich), sodium hydroxide (>98%, Sigma-Aldrich), potassium phosphate monobasic (98%, Sigma-Aldrich), and potassium phosphate dibasic (98%, Sigma-Aldrich).

Fabrication of gas diffusion electrode.

-Preparation of As-Purchased Cu NPs

The majority of experiments employed 25 nm Cu NPs unless otherwise noted. 25 mg Cu NPs were dispersed in 15 mL isopropyl alcohol and 75 µL Nafion 5 wt% and bath sonicated for 1 hour. The suspension was then sprayed onto carbon paper at 85°C with a spray-coater to achieve an active particle loading of 1 mg/cm^2. This loading, dispersion, and spraying procedure was employed for all Cu catalysts unless otherwise noted.

-Preparation of OD-Cu

OD-Cu was prepared by heating in a tube furnace at 500°C in air. The ramping speed was 5°C/min, the temperature was held for 1 hour, and ramp-down time was 5 minutes.

-Preparation of OD-Cu-Annealed

OD-Cu-Annealed was prepared following the procedure of Verdaguer-Casadevall et. al.[1] for the 350°C case. Briefly, the electrodes heated in a tube furnace in flowing N_2 for 2 h and cooled to room temperature in N_2.

-Preparation of Sputtered Cu

150 nm of Cu was sputtered on carbon fiber paper using an AJA Magnetron Sputter System using a high purity Cu target. The Cu was sputtered in 3 mTorr Ar at 100 W which corresponded to a measured deposition rate of 1 Å/s. The stage was rotating at 100 rpm throughout the duration of the deposition.

Photographs of these preparations are shown in **Figure S1**.

Fabrication of electrolysis cells

The fabrication of electrolysis cells with a 1 cm^2 active cathode and anode area is described here and shown in **Figure S2**. A cell with a 2 cm^2 active area was also made; the two types of cells differ only in the machine cuts for the cathode and anode chambers and in the length of the flow field.

Three approximately 2 x 2 x 0.5 in PEEK blocks (McMaster Carr) were cut using a band saw. Each block was then placed in a mill (Bantam Tools, Othermill) where the gas flow lines, liquid chambers, pilot holes where the screws are to be inserted were drilled, and the cell contours were cut. 1/8” bits were used to cut everything except the flow lines, and 1/32” bits were used to cut the flow lines. The cell was then flipped over and face cuts were used to cut the cell to thickness (1 cm). The cells were then sanded either manually or with a belt sander to remove any machine marks and to smooth the surface.

The pilot holes were then drilled completely using a 0.209” drill bit. Using a 0.043” drill bit, holes for the gas inlet and outlet were drilled at the ends of the flow lines. The panel containing the gas lines was then flipped and shallow holes were drilled at the same location where the gas lines were drilled using a 0.128” drill bit. 1/8” OD PEEK tubing was then cut into approximately 1 cm segments and inserted into the hole with epoxy as the adhesive.

For the cathode/anode chamber, a hole punch was used to make pilot holes above and below the chamber. A 0.128” drill bit was then used again to drill a hole for liquid flow. 2 cm segments of 1/8” OD PEEK tubing were then cut and inserted into the holes with epoxy as the adhesive.

On the catholyte chamber, on the center of another face, using a 0.063” drill bit, a thin hole was drilled for the reference electrode. Then a shallow hole was drilled in accordance to tap a ¼-28 hole, and the hole was then tapped using the ¼-28 tap. This is to fit a 1/8” flangeless PEEK fitting (Cole-Parmer), and a leakless Ag/AgCl reference electrode was inserted into this fitting.

A back plate was prepared in the same manner, except using a 0.25" thick PEEK block. Silicone gaskets were cut using a laser cutter or by hand. Long screws and wingnuts were used to hold the cell together.

See **Figure S2** for the geometry of the machined areas for the 1 cm^2 active area cell and for an overall schematic. Step files for the CAD drawings that were used to generate machine paths have been uploaded as additional supplementary materials.

Operation of electrolysis cell

The cells were assembled according to images in **Figure S2** using gaskets between every layer (6 total gaskets). The anode was Pt foil with Cu tape on the backside. Then the anolyte flow chamber and Selemion membrane were added. The catholyte compartment with the reference electrode was added. The reference electrode was saturated Ag/AgCl. Then the cathode was placed with the active Cu layer facing the catholyte compartment and Cu tape on the edge, so that feed gases could reach the back of the catalyst carbon paper. The cell top was then added so that the flow field had minimum change in vertical distance to minimize noise in transport. Cells were sealed using screws.

All experiments were conducted using 1M electrolytes of KOH unless otherwise noted. pH was tested using a calibrated pH meter system. pH 14 electrolyte was created by combining 1 L distilled water and 56 g potassium hydroxide (KOH). The procedure was identical for the 1M Na^+ and Cs^+ electrolytes. pH 6.3 electrolyte was created using 800 mL DI water, 95 g potassium phosphate monobasic, 52.5 g potassium phosphate dibasic, 200 mL DI water, and adjustment until pH=6.3. pH 8 electrolyte was created using 800 mL DI water, 162.82 g potassium phosphate dibasic, 8.878 g of potassium phosphate monobasic, and 200 mL DI water.

In some experiments, the electrolytes were pumped through the system using a peristaltic pump (MasterFlex, Cole-Palmer) at 1mL/minute. However, we discovered that the pressure pulses produced by the pump led to small fluctuation in the downstream mass flow measurements. As shown in **Figure S13**, use of a syringe pump for electrolyte supply greatly reduced this source of noise.

After electrolyte flow was established throughout the cell, the gas valve lines were opened. The valve line was connected to the bottom inlet of the cell so that the gas could travel vertically through the flow fields.

The cell was connected to a potentiostat (Biologic SP-300), and 5 rounds of cyclic voltammetry was conducted to reduce newly prepared OD-Cu between -1.1 V vs Ag/AgCl and -2.2 V vs Ag/AgCl. No further cyclic voltammetry was employed beyond this step or in between potentiostatic holds. After the cathode was reduced, each experiment involved an IR compensation step (ZIR) followed by potentiostatic holds. The experiments were conducted in different modes including: gas feed switching experiment with careful measurement of downstream mass flow, monitoring of gas phase products with gas chromatography instrument, and electrolysis to accumulate liquid products for NMR analysis.

Structural and electrochemical characterization and analytical chemistry

Structural characterization

Prepared samples were characterized using a scanning electron microscope (FEI Nova SEM 230) and X-ray diffractometer (Rigaku SmartLab). These characterizations are shown in **Figures S3-4.**

Detection of gas products

Gas chromatography (GC) was conducted according to the procedure described by Prabhakar *et. al.*[2] During electrolysis, CO was allowed to flow from the electrochemical cell directly into the gas sampling loop of a gas chromatograph for online gaseous product analysis. For all experiments, electrolysis proceeded for a minimum of 20 minutes with gas analysis sampling done after 8 minutes to fill the tube lines.

Detection of liquid products

Quantitative liquid product analysis was performed using ^{1}H NMR (Bruker Avance III 500 MHz spectrometer, 16 scans, 90% H_2O/10%, D_2O as lock solvent) and employing a procedure and analysis adapted from Chatterjee *et. al.*[3] The standard used was a solution of 50 mM phenol and 50 mM in DMSO. Each NMR sample was composed of 50 µL standard, 50 µL D_2O, and 400 µL electrolyte containing liquid products. Relaxation delay time d_1 was set to 25 seconds. Pulsecal (p1) and water peak (o1p) were determined for each electrolyte pH. The majority of experiments were performed with pH=14 with conditions were set to $p1_{KOH}$=18.820 µs and $o1p_{KOH}$=4.7291. The typical NMR analysis was performed on electrolyte aliquots taken after 10 minutes of potentiostatic electrolysis.

Calculation of Faradaic Efficiency for Gas and Liquid Products

Example GC and NMR data are shown in **Figure S5**. The faradaic efficiencies (FEs) are calculated using the same method (equations) as Singh *et. al.*[4]

NMR errors in faradaic efficiencies are attributed to error in electrolyte volume due to evaporation during storage. Errors in faradaic efficiencies can also come from incident gas leakage. Other reasons for variation are from slight sample-to-sample variation. For reservoir size determination, it is assumed that all product evolution was the same as the steady-state values; this will be discussed later in the SI.

Measurement of catalyst surface area

The electrochemical active surface area (ECSA) was derived from double-layer capacitance measurements. Cyclic voltammetry was performed between -0.2 V and -0.1 V vs Ag/AgCl with scan rates of 10, 25, 50, 75, 100, 150, 200, 250 and 300 mV/s. The double-layer capacitance was determined by the slope of the linear fits. The roughness factor was normalized to measurements on flat Cu foil to which we assigned a roughness factor of 1. The density of surface atoms, needed for the calculation of turnover frequencies (TOFs), was similarly scaled to the planar atoms/cm^2 density of Cu (**Table S1**).

This surface atom density calculation is assumed to be predominantly (111). This assumption is based on XRD pattern (**Figure S4**) and computational methods at relevant potentials (**Figure S6**). Common Cu orientations (100), (110), and (111) have planar densities of $15.3 \cdot 10^{14}$, $10.8 \cdot 10^{14}$, and $17.8 \cdot 10^{14}$ atoms cm^{-2}, respectively. The number of sites/cm^2 does not vary significantly for other Cu orientations. We assume a (111)-oriented surface when we calculate the reservoir size from the ESCA and delay time data. If other surface orientations were used in the calculation, it would not change the major findings of this work.

Computational Methods for Catalyst Orientation

Computational calculations were performed to understand the favored morphology of the Cu surface and aid ECSA determination. These calculations demonstrate the effect of applied potential on equilibrium surface faceting. They neglect any adsorbate interactions (CO is not considered in the equilibrium structures).

All DFT calculations were performed using VASP 5.4.4 employing the RPBE functional,[5,6] a plane-wave energy cutoff of 500 eV, and Brillouin zone sampling performed with a Monkhorst-Pack *k*-point density of 0.12 $Å^{-1}$. Symmetric surface slab models for facets up to

Miller index 3 were generated with the pymatgen code,[7,8] where all slabs exhibited lateral dimensions of at least 7 Å and thicknesses of at least 3 layers, with at least two top layers allowed to relax. The VASPsol implicit solvation method was used to model the charged interface and estimate potential-dependent surface energies.[9,10] VASPsol places countercharge by solving the linearized Poisson-Boltzmann equation, assuming monovalent point charges. The Debye length was set to 3 Å, corresponding to an ion concentration of 1 M, the dielectric constant was set to 80, and the surface tension (τ) was set to zero.

The surface energies of the Cu facets were determined from the electronic grand potential (Ω) of the clean surfaces as evaluated over a series of Fermi levels (μ_e) according to

$$\gamma^0_{hkl}(\mu_e) = \frac{1}{A_{hkl}}(\Omega_{hkl}(\mu_e) - NE_{Cu}) \quad \text{(S1)}$$

where N is the number of atoms in the slab of a given Miller index (*hkl*), E_{Cu} is the energy per atom in bulk fcc Cu, and A_{hkl} is the surface area of the slab. The energies were obtained following the methodology from Duan *et. al.*[11] The Fermi level μ_e was converted to potential on the SHE scale by

$$eU_{\mathrm{SHE}} = -\mu_e - 4.45 \quad \text{(S2)}$$

The potential-dependent surface energies of the *{hkl}* facets obtained from Eq. S1 were used to construct the Wulff shapes following the procedure in Refs. 9-10 for each constant Fermi-level (**Figure S6**).

Discussion of Catalyst and Microenvironment on Product Distribution

The spreadsheet "SI_Summary.xlsx" in the uploaded dataset documents performance and related data of various catalyst preparations under cell environments. It holds partial current densities densities calculated from EC-Lab, GC, and NMR, delay times, reservoir sizes, and turnover frequencies.

Figure S7 documents product distribution for OD Cu as a function of applied potential in pH=14 electrolyte.

Figure S8 documents faradaic efficiency as a function of applied potential and pH. We find that different catalyst pHs exhibit comparable C_2H_4 efficiencies at the same voltage on the SHE scale. Also notable is the effect that H_2 efficiency trends are reversed by higher pH. Specifically, at low pH (6.3, 8), HER appears more prominent at more negative applied potentials. This effect is reversed by high pH, as HER appears more prominent at more positive potentials.

Per **Figure S9**, with the increased catalyst defectiveness the partial current density to ethylene increases. We find that OD Cu has the best performance to ethylene based on its partial current density.

Gas feed switching

Experimental Goals

In this work, experiments track mass flow measurements (product evolution) based on controlled gas feed switching between inert Ar and feed CO. This is inspired by previous work with steady state isotopic kinetic analysis (SSITKA) and chemical transient kinetics (CTK). These experiments give insight into temporal concentrations of catalytically active phases (surface coverage of *CO on different sites) as they are built up or scavenged.

Gas feed switching with using GC injector valve

To the extent possible, we seek to make an abrupt switch between Ar and CO with minimal disruption to the gas flow characteristics of the system. To this end, we employed a run/vent method,[12] which we accomplished with a modified GC injection switching valve (Valco Cheminert C25Z-3186UMH). Compressed air to the switching valve (0-100 psig) was controlled by solenoid valves (Humphrey M31E1). The 12 VDC signal to operate the solenoid valves was provided by solid state relays controlled by digital signals generated by an Arduino microprocessor. Each 6 ports of the switching valve were connected to CO and Ar gas feed, flow cell and vent (**Figures S10-S11**).

Measurement of mass flow and gas feed switching

As precise measurement of the mass flow exiting the electrolysis cell with the highest possible time resolution is central to this work, we performed many control experiments to calibrate and quantify the response of the downstream mass flow meter (MFM).

Selection of appropriate MFM for switching experiment

We tested MFMs which operated based on thermal conductivity (MKS 0-10 sccm) and on measuring the pressure drop across an orifice (Alicat Series M 100SCCM). We found that time response of the former was too slow for useful measurements and thus employed the latter for all measurements in this work (**Figure S12**).

Modification of experiment for high precision data

Modifications were made to the setup to increase the precision of the data. Compared to peristaltic pumps, electrolyte syringe pumps were determined to cause less noise in mass flow measurements (**Figure S13**).

Modifications were made to the Alicat MFM to increase the data resolution **(Figure S14**). The provided software returns a mass flow reading at approximately 30 Hz; however, the values returned have only two significant figures after the decimal point, causing unnecessary truncation error. Instead, we used the analog output of the MFM which we digitized at 20 Hz with a high resolution (24 bit) ADC (NI 9219). A Python script was used to read the digitized ADC data via PyVisa using a wrapper around NIDAQmx.[13,14]

Response of the mass flow meter to changes in gas composition

The Alicat MFM employed in this work is also called a Coriolis mass flow meter, and it relies on the precise measurement of the pressure drop across an orifice and of the temperature of the gas.[15] The instrument first measures the differential pressure drop across the orifice, and then uses the Poiseuille Equation to calculate a volumetric flow rate:

$$\text{Volumetric flow} = \frac{(P_1 - P_2)\pi r^4}{8\eta L} = \frac{K\Delta P}{\eta}, \tag{S3}$$

where P_1 is the static pressure at the inlet, P_2 is static pressure at the outlet, r is the hydraulic radius of the restriction, L is the length of the restriction, K is a constant that encompasses the geometric factors of instrument, and η is the absolute viscosity of the fluid. The following equation is used to convert the volumetric flow to mass flow:

$$\text{Mass flow} = (\text{volumetric flow}) \frac{T_s}{T_a}\frac{P_a}{P_s}\frac{Z_s}{Z_a} \tag{S4}$$

where T_a is the absolute temperature of the fluid (measured by the MFM), T_s is the absolute temperature at standard conditions (STP), 273.15 K, P_a is the absolute pressure of the fluid (measured by MFM), P_s is the absolute pressure at standard conditions (STP), 1 atm, and Z_s/Z_a is a small correction for changes in gas compressibility between the measurement conditions and STP.

Consulting Eqns. (S3) and (S4), one can see that the MFM reading will depend on the gas viscosity. The MFM we employed was calibrated for CO. If the gas flow contains a component with a viscosity lower than CO (e.g. H_2), this will cause the reported mass flow to be lower than the true value. Similarly, gas composition containing gases with higher viscosity than CO

(e.g. Ar) will cause the MFM reading to be high. Response factors for the gases relevant to this work are tabulated in **Table S2**.[15]

Finally, we note that while we used an MFM for mass flow measurements in this work, use of other methods, such as rotameters, could be employed for cells with larger gas flow. Further, precise measurement of the change in mass flow produced by the electrolysis cell could be used in a similar way to assess feedstock conversion and the composition of the electrolysis products.

Response time of MFM reading to changes in mass flow

Changes in mass flow anywhere in the system (from the MFC to MFM) were near-instantaneously detectable by the downstream MFM, the relevant timescale being the speed of sound in 1 atm Ar or CO. In **Figure S15**, we conducted a MFM reading test by changing gas flow rate from 1 sccm to 2 sccm in upstream MFC (Alicat MC-100SCCM) and observing the response of MFM reading. The observed rise time, 0.045 seconds, corresponds well to the stated response times of the MFC (0.03 seconds) and MFM (0.01 seconds).

Time resolution

In addition to the instrumental time resolution discussed above, interdiffusion of the switched gases and the finite size of the cell will broaden the mass flow response, even in the limit of infinitely fast surface electrochemistry. We discuss the relative contribution of each mechanism here. The main effects we considered are interdiffusion of CO and Ar and the finite size of the cell. We should note that many prior chemical transient kinetics (CTK) experimental reports have not explicitly considered these broadening effects,[16–18] motivating us to do it here for our related approach.

Binary diffusion coefficients: Ar in Co and CO in Ar

Even for an infinitely abrupt change between Ar and CO, the boundary region where both gasses are present between the two gases will broaden as a function of time due to interdiffusion. We estimated the magnitude of this effect by first calculating the binary diffusion coefficients of Ar in CO and of CO in Ar. For calculating interdiffusion coefficients of each gas, we utilized an empirically determined equation from Fuller *et al.*[19]:

$$\widetilde{D} = \frac{1.00 \times 10^{-3}\, T^{1.75} \left(\frac{1}{M_{CO}} + \frac{1}{M_{Ar}}\right)^{1/2}}{p[{v_{CO}}^{1/3} + {v_{Ar}}^{1/3}]^2} \qquad \text{(S5)}$$

where $\widetilde{D}$ is the interdiffusion coefficient in units of cm^2/s, T is absolute temperature (K), M_{CO} and M_{Ar} are the molar masses of CO and Ar, p is the pressure in atm, and ν_{CO} and ν_{Ar} are tabulated "diffusion volumes" and are 18.9 and 16.1 at standard temperature and pressure, respectively. Using Eq. (S3), we obtained a $\widetilde{D}$ value of 0.1916 cm^2/sec at 298.15 K and 1 atm, which is comparable to D_{CO} and D_{Ar} in measured in air at the same conditions, which are 0.208 cm^2/sec and 0.189 cm^2/sec. This value is used in the Taylor-Aris dispersion analysis described below.

Taylor-Aris dispersion and effect of finite cell length

Due to the laminar flow velocity profile in the system, the interdiffusion will be faster compared to the static case. Within Taylor-Aris dispersion theory,[19–21] this is described by the dispersion coefficient K, which is related to the interdiffusion coefficient $\widetilde{D}$ as follows:

$$K = \widetilde{D} + \frac{R^2\underline{u}^2}{48D} \tag{S6}$$

where $\underline{u}$ is the mean flow velocity and R is the radius. For the conditions in this work K = 0.2074 cm^2 sec^{-1} at 1 atm, about 6% larger than $\widetilde{D}$. The Taylor-Aris dispersion theory assumes that the flow is laminar and fully developed, so the Reynold's number was calculated using the equation, $Re = \frac{\rho u D}{\mu}$ (D is tube diameter) obtaining values near 5, well within the laminar flow regime.

For visualizing the effect of interdiffusion, it is convenient to work in a moving coordinate system: $z^* = (z - \underline{u}t)$. Concentration profile for a pulse of gas A with mass M_A is

$$C(z^*, t) = \frac{M_A/\pi R^2}{\sqrt{4\pi K t}} e^{-\left(\frac{z^{*2}}{4Kt}\right)} \tag{S7}$$

Figure S16 shows the concentration profile of pulse of gas for typical conditions used in this work: at a mean velocity of 280 cm/minute and L_1 = 30.8 cm, t_1 = 6.60 s (dashed line).

We will now discuss how transient responses can be affected by our specific setup. We define transient responses to be instances when there are nonzero slopes in the mass flow measurements. Diffusional broadening takes approximately 1 second. Transit of the gas pulse through the flow field of the cell takes about 1.3 seconds. Convolving these values leads to the prediction that, in the limit of infinitely fast chemistry, the fastest mass flow response would be on the order of 1.5 seconds. Therefore, the resolution limit of the present set-up and cell size

is 1.5 s. Any transient response near or below 1.5 s cannot be accurately measured by this setup: for example, if a response measures 1.5 s, the underlying chemical process may be much faster.

Measurement of gas velocity

Equation (S8) relates the mass flow rate and volumetric flow rate. The mean velocity $\underline{u}$ is obtained using the cross-sectional area of the tubing:

$$\underline{u} = \frac{volumetric\ flow\ rate}{\pi r^2} \tag{S8}$$

where r is the radius of the tubing. The ID of the 1/16" diameter tubing we employed is stated by the manufacturer to be 0.03125 in (0.079375 mm). Use of Eqs. (S3) and (S8) at 298.15 K yields a mean flow velocity of 4.60 cm/sec (276 cm/min).

In this section, we aimed to measure gas velocities through tubing connecting the MFM and cell by switching the gas from CO to Ar and monitoring the arrival time of the switched gas using the MFM. The procedure involved maintaining a constant flow of CO at 5 SCCM for 30 seconds, after which the gas feed was switched to Ar. The time of rapid change in the MFM value was recorded to determine the gas arrival time. This procedure was repeated for tubes of 50.5 cm, 101.0 cm, 151.6 cm, 202.1 cm, 252.6 cm and 303.1 cm to investigate the influence of tube length on gas arrival time.

Table S3 presents theoretical and experimental onset times based on the tube length. Here, the onset time is defined as the time taken for the new feed gas (Ar) to reach the MFM after a switch (from CO to Ar). The 5 sccm onset time column assumes there is no interdiffusion between Ar and CO, with the gases traveling at 5 sccm consistently. The interdiffusion onset time column accounts for the assumption of active interdiffusion between Ar and CO upon switching the gas inlet, incorporating an additional diffusion length of $2\sqrt{\tilde{D}t}$ to the tube length. In **Table S3**, the experimental onset time column represents the actual onset time observed in our experiments.

Considering the possibility of active interdiffusion between Ar and CO, an increase in onset time is expected, as gases will interdiffuse into each other while traveling. In agreement with this expectation, both the interdiffusion onset time and experimental onset time are greater than the 5 sccm onset time. The experimental onset times exceed the interdiffusion onset times due to the dead volume in the MFM inlet of 0.0126 cm^3, because the gas must travel a greater distance before detection by the MFM. Although this factor impacts the time resolution of

measurements, it does not affect the measurements presented in the main text, as the composition of the gas mixture transiting the MFM remains unchanged during the relevant measurement period (t_{delay} + $t_{transition}$).

Estimate of transit time of gas through carbon paper

We considered the possibility that the transit time of the gas flow through the carbon paper would affect the timing of the gas supply to the catalysts. To calculate the transit time through the porous structure of the carbon paper, we followed the approach of Choi *et al.*[22]:

$$D_{CP} = D_i \frac{\epsilon}{\tau} \quad \text{(S9)}$$

where D_{CP} is the effective diffusion coefficient in the carbon paper, D_i is the self-diffusion coefficient of the gas, and τ represents tortuosity associated with carbon paper, and ϵ is porosity of carbon paper. To obtain the tortuosity value τ for our carbon papers we used the approach of Amini *et al.*[23]:

$$\tau = \left(\frac{\epsilon - \epsilon_p}{1 - \epsilon_p}\right)^{-\alpha} \quad \text{(S10)}$$

where, ϵ_p is percolation threshold of randomly overlapping fiber structures of various directionalities, and α is a computationally determined exponent for the equation above. For our model, we utilized the values of $\epsilon_p = 0.11$ and $\alpha = 0.521$. We were able to obtain the porosity values from specs sheets of carbon papers. For the percolation threshold and the corresponding exponent, we obtained such values from Tomadakis *et al.*[24] In order to simplify our calculation, we assumed that all gases travelled through the carbon paper orthogonally. In the experiments conducted in the main text, we utilized carbon papers with thicknesses of 215 μm, 325 μm and 420 μm with porosities of 0.8, 0.8 and 0.82, respectively.

For CO, using the values, for carbon paper with thickness 215 μm, we obtained τ value of 1.14 and D_{CP} value of 0.146 cm^2/sec. For carbon paper with thickness of 325 μm, we obtained $a\ \tau$ value of 1.14 and D_{CP} value of 0.146 cm^2/sec. Lastly, for carbon paper with thickness of 420 μm, we acquired a τ value of 1.12 and D_{CP} value of 0.140 cm^2/sec. For Ar, using the values, for carbon paper with thickness 215 μm, we obtained a D_{CP} value of 0.132 cm^2/sec. For carbon paper with thickness of 325 μm, we obtained a D_{CP} value of 0.132 cm^2/sec. Lastly, for carbon paper with thickness of 420 μm, we acquired D_{CP} value of 0.140 cm^2/sec.

Now, we can calculate the transit time of Ar and CO through different thicknesses of carbon papers, employing the computed D_{CP} values. For such calculation, we assumed that the diffusion type is analogous to that of Fick's Second Law since there is no driving force other than the Brownian motions of the molecules and their concentration gradient, eliminating the need for advection term. Thus, for the gas concentration C we have:

$$\frac{\partial C}{\partial t} = D_{cp}\frac{\partial^2 C}{\partial x^2} \tag{S11}$$

with boundary conditions of $C(x,0) = 0$, $C(\infty,t) = 0$ and $C(0,t) = 0$, in which we assume it is a semi-infinite medium. The analytical solution to Eq. (S11) is:

$$C(x,t) = C_0\, erfc\left(\frac{x}{2\sqrt{D_{cp}t}}\right) \tag{S12}$$

which gives the diffusion length of $2\sqrt{D_{CP}t}$. We can equate the diffusion length equation with the carbon paper thicknesses and acquire the transit time as well.

Ultimately, we attained the transit times of 3.5×10^{-3} sec, 8.0×10^{-3} sec and 1.5×10^{-2} sec for carbon paper thicknesses of 215 μm, 325 μm and 420 μm, respectively. Importantly, these transit times are significantly shorter than the time scales we are observing. We conclude that the finite transit time of the gases through the carbon paper will not affect our measurement.

Experimental measurement of delay time

Definitions of Important Timescales: Arrival, Delay, Transition, and Onset Times

This experiment is rich in temporal information. We define various timescales to clarify experimental findings, shown in **Figure S17-18**.

Arrival time is taken to be the time elapsed between a gas feed switch and **the arrival of the new gas to the *catalyst cell.*** Typical experiments were conducted using the system of L_1 = 30.83 cm, L_{cell} = 6.066 cm, L_2 = 111.68 cm and $\underline{u}$ = 4.60 cm/s. Using these tube lengths, the arrival time is 6.60 s. This tells us that we cannot learn any information in the first 6.60 s after the switch because no new chemistry is occurring on the catalyst.

Delay times are defined as the difference between the arrival of a new gas and the subsequent 1% change from steady state mass flow conditions. These are described in **Figures S17-18**. This gives information about how long the local environment of the catalyst stays the same before reactants are then scavenged or built up on the surface. Delay times are the most

important metric in this work because they give information about the underlying chemistry and timescale for *CO atoms to start to react or desorb from the Cu surface.

Transition times are used to understand the duration of a nonzero slope in mass flow measurement. In this work, transition times are used to characterize the resolution limit of the MFC and MFMs (**Figure S19**). Future work can use transition time to consider scavenging or build-up behaviors and possible insights into reaction rate constants and diffusion.

Onset times are used to understand the time elapsed between a gas feed switch and **the arrival of a new gas to the *mass flow meter*.** Thus, onset times must be larger than arrival and delay times for information about underlying reaction chemistry to be available. For example, Figure **S20** demonstrates that the onset time in this work is approximately 40 s.

Temporal Insights from Mass Flow Measurements and Potentiostat Data

Mass flow measurements and potentiostat data (currents in mA) are well-coordinated in time as shown in overlays of MFM and EC-Lab data in **Figure S21**. Thus, these data are complementary and characterize the same conclusions. We utilize delay time from the MFM measurement because this work aimed to investigate chemical transient kinetics. We emphasize that currents tell us information about charge transfer but do not clarify which reactions occur. The mass flow measurements yield more important information about the relative prevalence of various reactions (C-C coupling has lower outlet flow rates than those of HER).

Control Experiments and Microenvironmental Effects

A myriad of control experiments was performed. Some experiments have already been discussed relating to mass flow simulations and response times. **Figures S22-23** demonstrate the negligible effects of tube length and carbon paper thickness on delay time phenomena. These corroborate that the delay time is intrinsic to the catalyst microenvironment and is due to a local reservoir, rather than dead volume. Further experiments show negligible effects of ionomer and mass loading on delay time phenomena in **Figures S24-25**.

Noticeable effects that affected the observed delay time include those discussed in the main text (catalyst preparation, partial pressure, electrolyte cation, and pH) and mass loading. We show raw MFM data for the effect of cation size (**Figure S26**) to demonstrate the different MFM signatures as a function of environment which tells possible differences in product

distribution. We show that the nanoparticle size (**Figure S27)** also has an effect on the catalyst delay time, though this result requires further investigation.

Reservoir Size and Turnover Frequency Calculation

We calculate reservoir size and turnover frequency to determine the heterogeneity and activity of the Cu surface for various preparations and microenvironments. For these calculations, the electrochemical surface area (*ECSA*) is used assuming a (111) structure with no reconstruction nor oxide formations during operation. Steady state faradaic efficiencies and current densities will be used in the calculations. This assumption is reasonable because mass flow measurements are very similar during the delay time and during the steady state.

First, we determine the CO reduction rate to ethylene via potentiostat data and GC faradaic efficiencies. These determinations are based on Equations S10 and S11, where $J_{C_2H_4}$ is partial current density to ethylene, *FE%* is faradaic efficiency from GC, and *F* is Faraday's constant.

$$J_{C_2H_4} = FE\% \times J_{TOT} \quad \text{(S13)}$$

$$CORR = J_{C_2H_4} \times 2/(8 * F) \quad \text{(S14)}$$

Then, we can determine the reservoir size from delay time measurements (CTK experiment):

$$Reservoir\ Size = CORR \times Delay\ Time \quad \text{(S15)}$$

$$Reservoir\ Coverage = Reservoir\ Size/ECSA \quad \text{(S16)}$$

The turnover frequency (*TOF*), taken to be the turnover frequency of non-reservoir sites, and its standard deviation $\delta(TOF)$ is calculated from equations sx and sxx.

$$TOF = CORR \times (1 - Reservoir\ Fraction) \times ECSA \quad \text{(S17)}$$

$$\begin{aligned} \frac{\delta(TOF)}{TOF} &= \sqrt{\left(\frac{\delta(CORR)}{CORR}\right)^2 + \left(\frac{\delta(1 - Reservoir\ Fraction)}{1 - Reservoir\ Fraction}\right)^2} \\ &= \sqrt{\left(\frac{\delta(FE\%)}{FE\%}\right)^2 + \left(\frac{\delta(1 - Reservoir\ Fraction)}{1 - Reservoir\ Fraction}\right)^2} \end{aligned} \quad \text{(S18)}$$

Notably, these turnover frequencies are "combined turnover frequencies" because multiple active sites can contribute. When we discuss reservoir, terrace, and defect sites we assume that this turnover frequency arises as a combination of terrace and defect sites.

Turnover frequencies are documented in the main text and the attached SI_Summary.xlsx spreadsheet. They depend on the operational conditions of the reactor and should be compared at a constant voltage vs. SHE to have the same driving force for activity.

Figure S28 shows the positive relationship between partial current density to ethylene vs. reservoir coverage using different preparations at the same overpotential. **Figure S29** shows the positive relationship between combined TOF to ethylene vs. reservoir coverage. For various nanoparticle preparations at the same overpotential, higher reservoir coverages also have higher combined TOF. We also demonstrate that this relationship might transcend applied potential for OD Cu, as the functional relationship between reservoir coverage and combined TOF looks exponential.

Microkinetic model

Rationale and Setup of Microkinetic Model

We sought to find the simplest possible model that can explain why certain Cu preparations lead to delay times. Delay times are when the COR reaction continues at the same rate as measured by both current density and reactor mass flow despite there being no new supply of CO. The oxide-derived Cu (OD Cu) nanoparticles have experimentally observed delay times ranging from 1.5 to 7 seconds with corresponding reservoir sizes from 10^{-9} to 10^{-7} mol/cm^2. In contrast, the as-received Cu nanoparticles do not appear to show this reservoir effect.

Here, we employ multi-site models that include diffusion and C-C coupling reactions. Each catalyst site has different binding energies and C-C coupling rates in accordance with our own DFT calculations and previous characterizations from literature. We found that a 2-site model with 1 active site and 1 inactive site can explain a delay time. However, the 2-site model results in diffusion constants and reaction rates that are unphysical. We thus proposed a 3-site model that encompasses physical motifs and leads to a reasonable explanation of delay time.

Each model involves reaction rates dependent on the coverage of CO: each site can either be empty or occupied by 1 CO molecule. For the ith reaction, we define the forward rate constant to be k_{+i} and the reverse rate constant to be k_{-i}. The reaction rates v_i are calculated from the instantaneous surface coverages ($\theta^{CO}_{site\ type,}\theta^{Vacant}_{site\ type}$), the rate constants ($k_{+i}, k_{-i}$), and the density of surface sites (Γ_s). Current densities are calculated based on the formation of C-C products; we assume these products to be ethylene. We select reaction and diffusion rates and impose initial conditions of surface coverages.

ODEs were solved numerically as no analytical solution exists. Specific details are available in the “two_site_model.ipynb” and “3_site_model.ipynb” notebooks.

Microkinetic Model Assumptions

Reasonable assumptions were imposed to ensure the most simplistic microkinetic model. Firstly, CO is assumed to be consumed irreversibly. The system will not be replenished with CO during the simulation. Thus, CO will only decrease in concentration. The simulation also assumes that the only reactions are C-C coupling: no hydrogen evolution is modeled and no competing reactions to other products are considered. This approach is an initial value problem that simulates CO coverage just prior to an experimental switch. Thus, this model is ineffective at modeling steady state conditions.

This model assumes site conservation: no site will lose activity over time, nor will poisoning/blocking of available sites occur other than short-lived CO molecules coverage. This model also assumes that C-C products desorb quickly or have desorption rates embedded in the reaction constants.

Once the simulations begin, we define the delay time metric to occur when 90% of the current density has been attenuated. The qualitative trends from these simulations can thus be compared to experimental results.

3-Site Model

The 3-site model is discussed in the main text. Relevant diffusion and reactions are shown below.

$$\theta_{Reservoir}^{CO} + \theta_{Terrace}^{Vacant} \leftrightarrow \theta_{Reservoir}^{Vacant} + \theta_{Terrace}^{CO}\ (k_1, k_{-1}) \qquad \text{(S19)}$$

$$\theta_{Reservoir}^{CO} + \theta_{Defect}^{Vacant} \leftrightarrow \theta_{Reservoir}^{Vacant} + \theta_{Defect}^{CO}\ (k_2, k_{-2}) \qquad \text{(S20)}$$

$$\theta_{Terrace}^{CO} + \theta_{Defect}^{Vacant} \leftrightarrow \theta_{Terrace}^{Vacant} + \theta_{Defect}^{CO}\ (k_3, k_{-3}) \qquad \text{(S21)}$$

$$2\theta_{Reservoir}^{CO} \rightarrow 2\theta_{Reservoir}^{Vacant} + C2Product\ (k_4) \qquad \text{(S22)}$$

$$2\theta_{Defect}^{CO} \rightarrow 2\theta_{Defect}^{Vacant} + C2Product\ (k_5) \qquad \text{(S23)}$$

$$2\theta_{Terrace}^{CO} \rightarrow 2\theta_{Terrace}^{Vacant} + C2Product\ (k_6) \qquad \text{(S24)}$$

$k_{1,2,3}$ and $k_{-1,-2,-3}$ are the diffusion rate constants and $k_{4,5,6}$ are the C-C coupling rate constants. The chosen values of these rate constants are provided in Table 1 in the main text.

Below we show the sensitivity of the delay time profiles on the diffusion rate constants and the C-C coupling rates for $\theta_{*CO,r} = 0.71, \theta_{*CO,t} = 0.07, \theta_{*CO,d} = 0.21$ that corresponds to a situation where we observe a delay time profile similar to experiments. We independently vary the values in Table 1 by an order of magnitude and observe its influence on the delay time profiles.

From the profiles in **Figures S30 and S31**, we find that the delay time profiles are sensitive to:

(i) k_2 and k_{-2} the diffusion rate constants for *CO from reservoir and defect sites: Slower diffusion from reservoir to defect sites compared to the value in Table 1 results in disappearance of the delay time as the reservoir sites are not able to supply *CO fast enough to the defect sites to sustain the current density. Similarly, faster diffusion from the defect to reservoir sites compared to Table 1 will also result in the disappearance of the delay time.

(ii) k_4, the C-C coupling rate constant for the reservoir sites: Faster rates for C-C coupling on the reservoir sites also results in a disappearance of the delay time as the reservoir *CO is being consumed by the C-C coupling reaction instead of diffusion to the defect sites needed to observe the delay time.

(iii) k_5, the C-C coupling rate constant for the defect sites: Faster rates for C-C coupling on the defect sites results in disappearance of the delay time as the *CO is being consumed at a faster rate than the reservoir sites can supply *CO to the defect sites.

Diffusion Distances Calculation

In the case of long delay times, one might wonder how far a CO molecule may move on the surface. Therefore, we introduce methods to estimate (1) the average distance traveled by the CO on the surface, (2) its relation to nanoparticle size, and (3) number of times a CO molecule may encounter an active site.

To do these calculations, diffusion is assumed to be mediated by a 2-dimensional catalyst surface. Diffusion is also assumed to be isotropic. Further, we note that the diffusion constants for CO on Cu in electrolyte is difficult to measure and has not been done by us nor previous

works. Thus, we assume that diffusion constants for CO on Cu in electrolyte can be approximated by the diffusion constants for CO on Pt in electrolyte.[25] These diffusion constants are functions of surface coverage.

Detailed Diffusion Calculations

For 2-dimensional diffusion, the maximum diffusion length $L_{traveled}$ is given by Eq. S19, where *t* is the delay time and *D* is the diffusion constant. This diffusion length is the distance CO might travel.

$$L_{traveled} = \sqrt{4D \cdot t} \tag{S25}$$

The distance traveled can be compared to this work's typical nanoparticle size (25 nm per manufacturer). To make this comparison, we assume that active sites are homogeneously dispersed to have an average active site spacing ($d_{Active-Active}$) inversely proportional to the fraction of active sites (θ_{Active}^{CO}). The proportionality constant is the interplanar spacing of the (*hkl*) plane (d_{hkl}). This relationship is shown in (Eq. S20).

$$d_{Active-Active} = \frac{d_{hkl}}{\theta_{Active}^{CO}} \tag{S26}$$

The average number of times a CO molecule may encounter an active site is given by Eq. S27.

$$Number\ Encounters = \frac{L_{traveled}}{d_{Active-Active}} \tag{S27}$$

With these approximations, we choose 2 representative calculations (**Table S4**) using the minimum diffusion constant corresponding to completely covered surface and maximum diffusion constant corresponding to 0 surface coverage. These calculations demonstrate trends or orders of magnitude for diffusion during the delay time. Based on **Table S4**, CO may travel far distances, comparable to the particle diameter, and encounter multiple active sites. Continuing this logic, other Cu preparations (sputtering, as purchased) likely have significantly lower CO surface diffusion distances.

Supplemental tables

Table S1. ECSA measurements and corresponding estimated surface site density of Cu. It is assumed that Cu atoms are arranged in the (111) orientation. These measurements are taken after standard electrode conditioning (5 CV scans). ECSA was from cyclic voltammetry performed between -0.2 V and -0.1 V vs Ag/AgCl with scan rates of 10, 25, 50, 75, 100, 150, 200, 250 and 300 mV/s.

Sample	Electrolyte	Capacitance (mF)	Roughness factor	Cu atoms on surface (mol/cm^2)
Flat Cu foil	1 M KOH	0.089	1	2.94e-9
Sputtered Cu	1 M KOH	8.38	94.2	2.77e-7
OD-Cu NPs (40 nm)	1 M KOH	13.1	147	4.43e-7
OD-Cu NPs (25 nm)	1 M KOH	22.8	256	7.54e-7
OD-Cu NPs (25 nm)	0.70 M KH_2PO_4,<br>0.30 M K_2HPO_4	4.59	51.6	1.52e-7
OD-Cu NPs (25 nm)	0.07 M KH_2PO_4,<br>0.93 M K_2HPO_4	4.97	55.8	1.65e-7
Cu NPs (40 nm)	1 M KOH	9.85	111	3.26e-7
Cu NPs (25 nm)	1 M KOH	14.7	165	4.85e-7

Table S2. Calibration factors for MFM readings relative to CO. As an example, a 5 sccm Ar flow will produce a reading of 6.41 sccm (5*1.282) on an MFM calibrated for CO.

Gas	μ (1e-5 Pa-sec)	Response Relative to CO
Ar	2.23	1.282
N_2	1.76	1.011
CO	1.74	1.000
CO_2	1.47	0.845
CH_4	1.10	0.632
C_2H_4	1.03	0.592
H_2	0.88	0.506

Table S3. Comparison of predicted and measured onset times for different tube lengths and volumes. Here, the onset time is defined as the time taken for Ar to first reach the MFM. Third column, 5 sccm onset time (s), calculates the onset time with the assumption of no interdiffusion between Ar and CO; fourth column, interdiffusion onset time (s), calculates the onset with the assumption of interdiffusion between two gases; the last column, experimental onset time (s), represents the actual onset time we observed. The small difference between the fourth and fifth columns is due to the small dead volume in the MFM. Notably, the error between calculated interdiffusion and experimental onset times is less than 4%.

Tube volume (mL)	Tube Length (cm)	5 sccm Calculated onset time (s)	Calculated Interdiffusion onset time (s)	Experimental onset time (s)	Error (%)
1	50.5	12.0	12.7	13.2	-3.79
2	101.0	24.0	25.0	25.4	-1.57
3	151.6	36.0	37.3	37.5	-0.53
4	202.1	48.0	49.5	49.2	0.61
5	252.6	60.0	61.6	62.2	0.96
6	303.1	72.0	73.8	74.4	0.81

Table S4. Diffusion Calculations for a delay time of 4 seconds and reservoir fraction of 0.8. This most closely resembles OD-Cu at -0.75V vs RHE in pH=14 KOH, which has a delay time of 3.9 seconds and reservoir fraction of 0.78. Only 1 significant figure should be trusted from the results, but all are shown for clarity of the model's assumptions. The small estimate uses the diffusion constant of CO on Pt at 100% surface coverage and (100) interplanar spacing. The large estimate uses a faster diffusion constant of CO on Pt at 0% surface coverage and a smaller (111) interplanar spacing.

Case	Diffusion Constant (cm^2/s)	Diffusion Length (nm)	Ratio of diffusion length to nanoparticle diameter (-)	d_{hkl} (nm)	$d_{Active-Active}$ (nm)	Number Active Site Encounters
Small Estimate	3.6e(-13)	24	0.96	0.362	1.81	13
Large Estimate	2.2e(-12)	59	2.0	0.209	1.04	57

Supplemental figures

Figure S1. Photographs of Cu Electrode Preparations
Figure S2. Scheme and dimension of GDE flow cell.
Figure S3. SEM images of preparations of OD-Cu NPs and Cu NPs prior to use.
Figure S4. XRD spectra of preparations of OD-Cu NPs and Cu NPs prior to use.
Figure S5. Example of Quantitative Product Analysis (GC and NMR).
Figure S6. Equilibrated Wulff Constructions: voltage-dependent area fraction of (100) and (111) planes on clean Cu, assuming no CO nor other adsorbates interact.
Figure S7. Changes in OD Cu catalyst performance with change of applied potential
Figure S8. Faradaic efficiencies towards ethylene for OD Cu at various pH.
Figure S9. Product distributions as a function of Cu preparation.
Figure S10. Operation switching valve of Valco Cheminert C25Z-3186UMH.
Figure S11. Photo images of GDE flow cell and gas switching valve.
Figure S12. Importance of Pressure-Drop Mass Flow Meter for Improved Transient Resolution.
Figure S13. Reduction of noise in the MFM reading via use of syringe pump.
Figure S14. Mass flow measurement with improved digitization.
Figure S15. Instantaneous Response Times of Mass Flow Measurements.
Figure S16. Simulation of Diffusional Broadening of Delta-Function Impulse of concentration.
Figure S17. Overall switching experiment from CO to Ar to CO.
Figure S18. Determination of Delay Time.
Figure S19. Characteristic mass flow measurements with repeatedly applied on/off potential.
Figure S20. Mass flow measurements without catalyst nor applied potential.
Figure S21. Current density profile of overall switching experiment.
Figure S22. Control experiment of delay time with change of tube length
Figure S23. Control experiment of delay time with change of C paper thickness.
Figure S24. Control experiment of delay time with change of ionomer
Figure S25. Control experiment of delay time with change of mass loading of OD Cu NP catalyst.
Figure S26. Effect of different cation size of electrolyte on mass flow measurement
Figure S27. Effect of OD Cu original nanoparticle size on delay time.
Figure S28. Partial current density to ethylene as a function of *CO reservoir coverage.
Figure S29. Evidence that *CO reservoir coverage increases combined turnover frequency to ethylene
Figure S30. Effect of diffusion constants on current density and delay time
Figure S31. Effect of C-C coupling rate constants on current density and delay time.

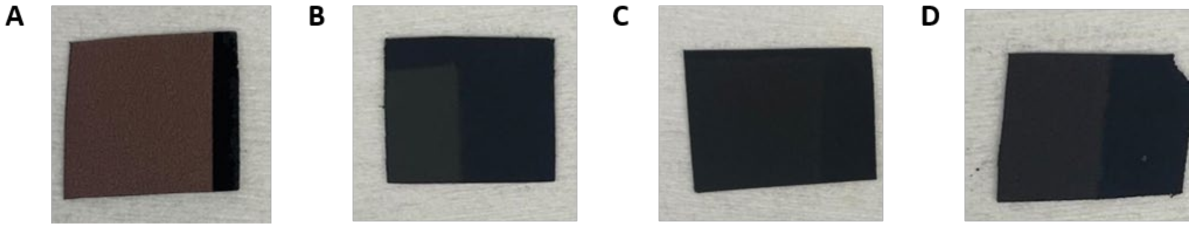

Figure S1. **Photographs of Cu Electrode Preparations** prior to use. (A) sputtered Cu, (B) as-purchased Cu NPs, (C) OD-Cu NPs, and (D) OD-Cu NPs annealed. The cathodes in these images are approximately 1x1 cm^2 and the right sides are the carbon paper.

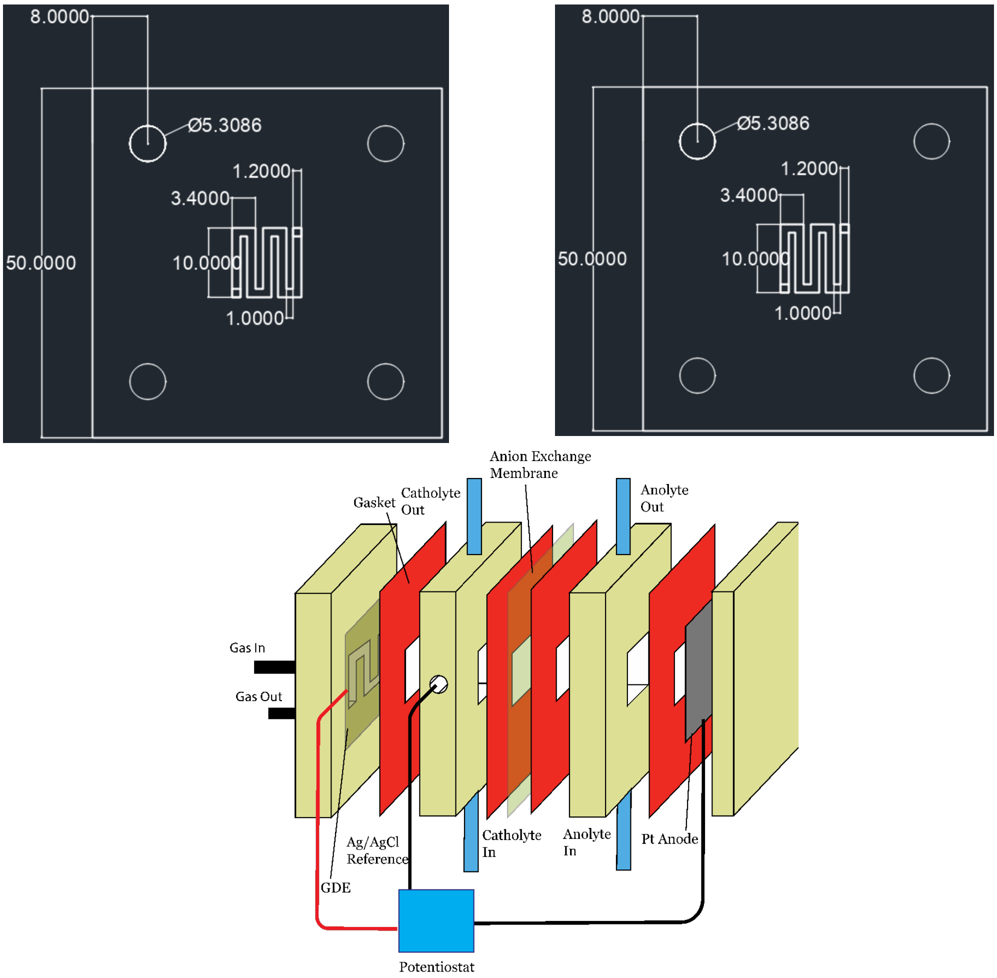

Figure S2. **Scheme and dimension of GDE flow cell.** (A) Scheme and dimensions of the flow field panel of the 1 cm^2 GDE cell. (B) Scheme and dimensions of the cathode/anode chamber of the 1 cm^2 GDE cell. (C) Schematic of the fully assembled GDE cell. All values are in mm.

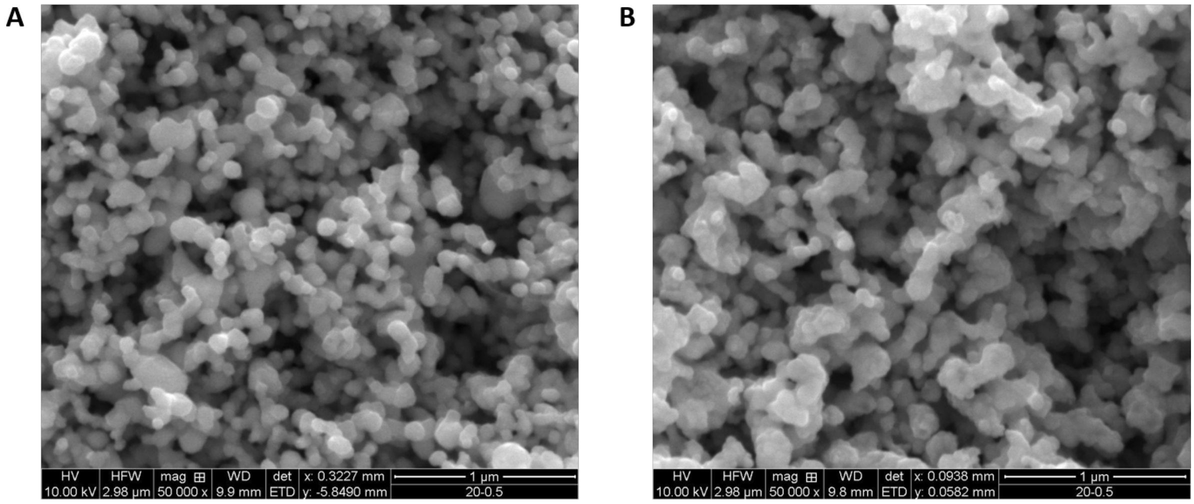

Figure S3. **SEM images of preparations of OD-Cu NPs and Cu NPs prior to use**. Spray-coated (A) as-purchased Cu NPs and (B) OD-Cu_NPs on GDE.

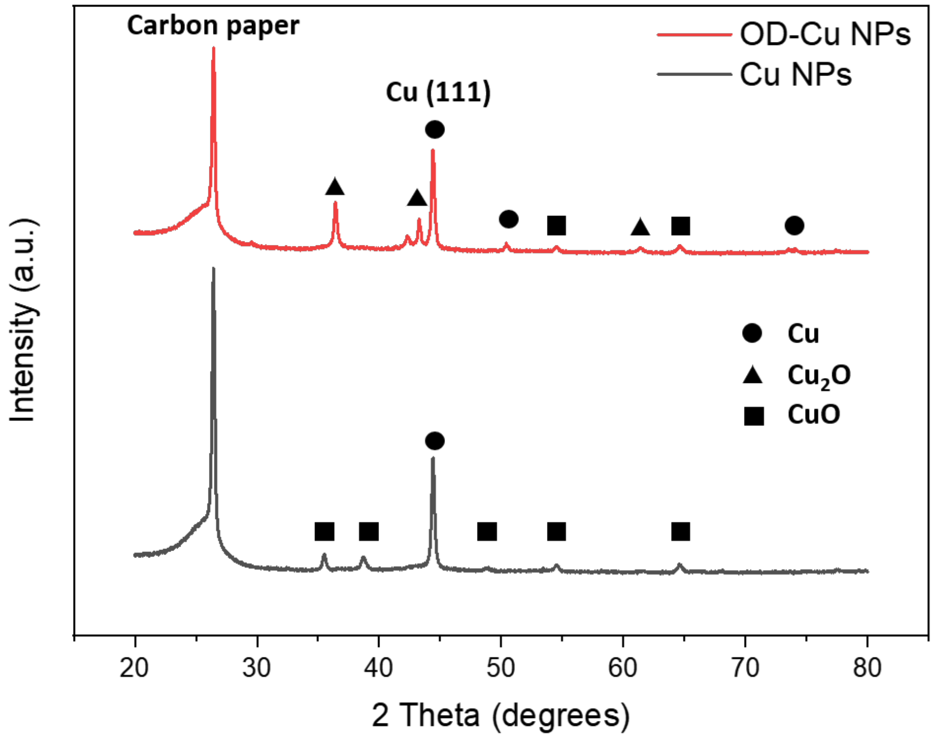

Figure S4. XRD spectra of preparations of OD-Cu NPs and Cu NPs prior to use. This demonstrates that OD-Cu NPs have significant Cu_2O and CuO. Cu NPs have some CuO. This also demonstrates that the (111) plane is most dominant after preparation.

a)

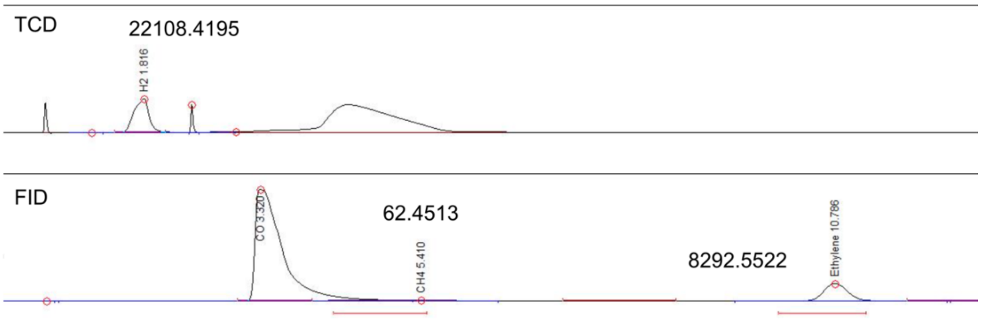

b)

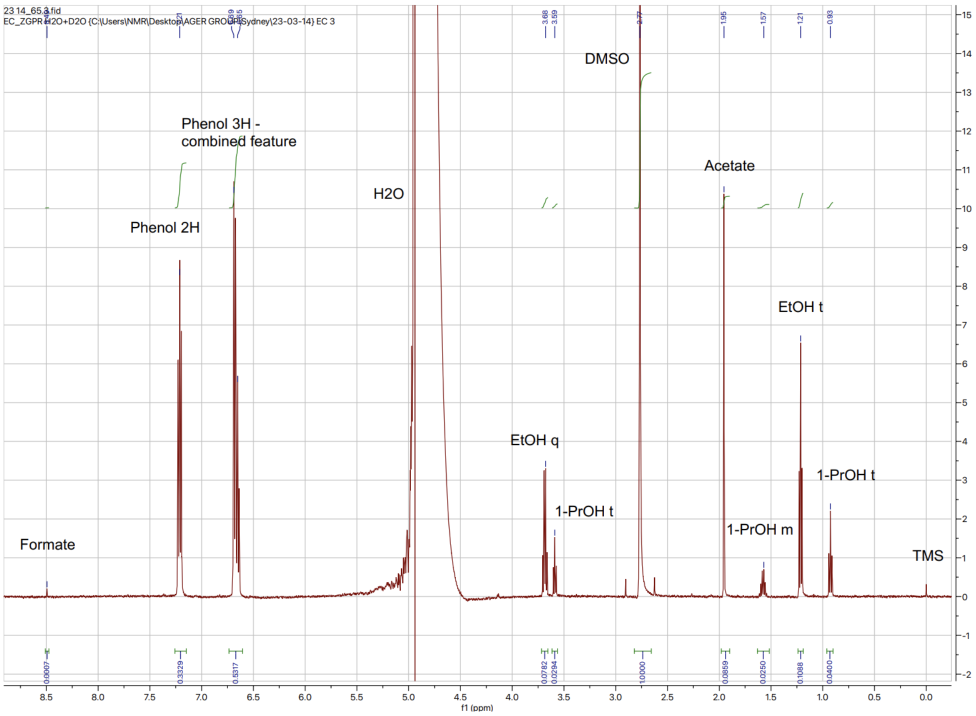

Figure S5. Example of Quantitative Product Analysis (GC and NMR). A) These spectra (TCD and FID) are taken from Fumion/OD-Cu -1.47 V vs. SHE in 1M KOH with injection after 8 minutes of running. Time elapsed (minutes) are labeled vertically and ppm measurements are labeled horizontally. CO ppm measurements are not used because it is the flow gas. **B)** This spectrum is taken from Nafion/OD-Cu -1.47 V vs. SHE in 1 M KOH after potentiostatic run for 10 minutes. Peaks are labeled in black according to product and NMR peak type (for example, t = triplet). TMS stands for tetramethylsilane. Blue numerical annotations above are for product peak locations, and blue annotations below exemplify integration areas. Both FE% can be found in **Figure S7.**

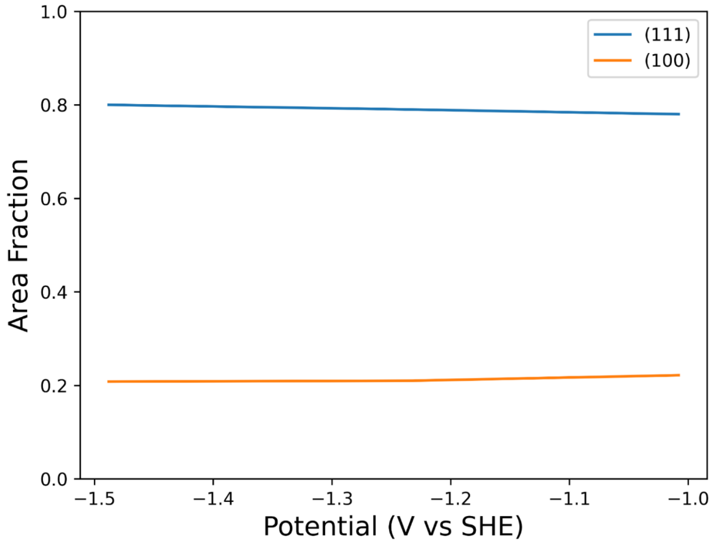

Figure S6. Equilibrated Wulff Constructions: voltage-dependent area fraction of (100) and (111) planes on clean Cu, assuming no CO nor other adsorbates interact. This demonstrates that the (111) orientation is expected to dominate all operating potentials in this study. The (110) plane has negligible fractions at the operating potentials employed in this paper.

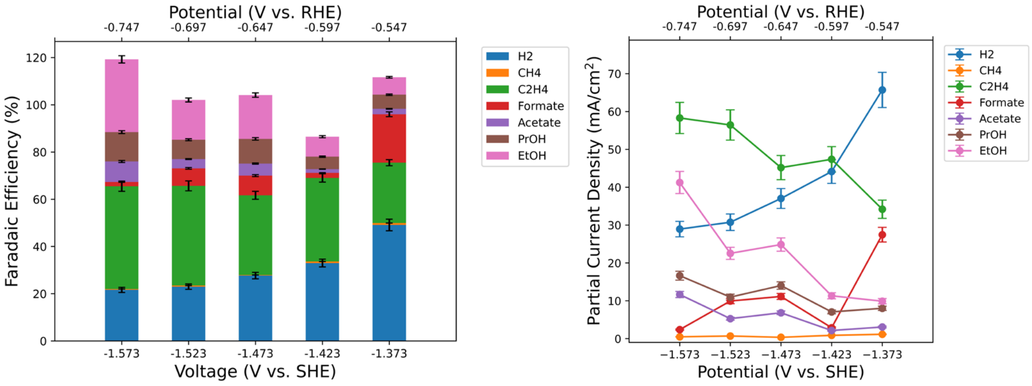

Figure S7. **Changes in OD Cu catalyst performance with change of applied potential**. A) Typical faradaic efficiencies for the catalyst system in this work. Notably, C2+ products dominate. Because most charge goes towards multicarbon products, the microkinetic models only consider C-C coupling reactions. B) Partial current densities of each product. System conditions are 25 nm OD-Cu and Nafion on C paper, 1 M KOH, 1 mL/min electrolyte, 5 sccm gas flow rate, and partial CO pressure of 1 during the switch. One may note that there are some kinks in the data, which can be due to sample-to-sample variation. Error bars are taken from the same sample's faradaic efficiencies and total current density variation.

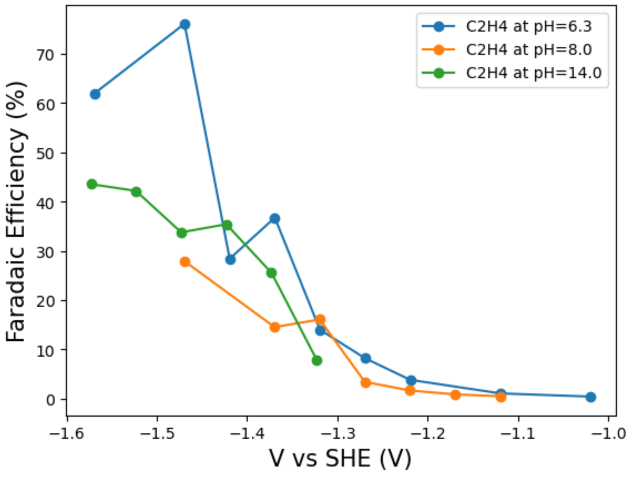

Figure S8. Faradaic efficiencies towards ethylene for OD Cu at various pH. Notably, efficiency towards ethylene is similar at the same voltage on the SHE scale.

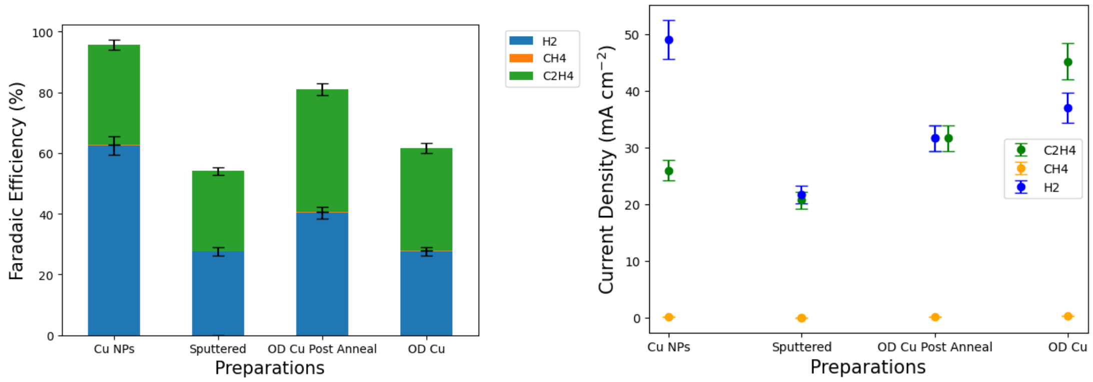

Figure S9. Product distributions as a function of Cu preparation. Left: Faradaic efficiency. Notably, the largest observed faradaic efficiency towards ethylene at this potential is achieved by OD Cu Post Anneal. Right: Partial current density towards gas-phase products. The partial current density to ethylene matches trends of defectiveness: increasing from Sputtered Cu, Cu NPs, OD Cu Post Anneal, and OD Cu. OD Cu stands for OD Cu dispersed on Nafion. All preparations were tested in 1 M KOH using steady state -1.47 V vs. SHE with 1 bar CO flow.

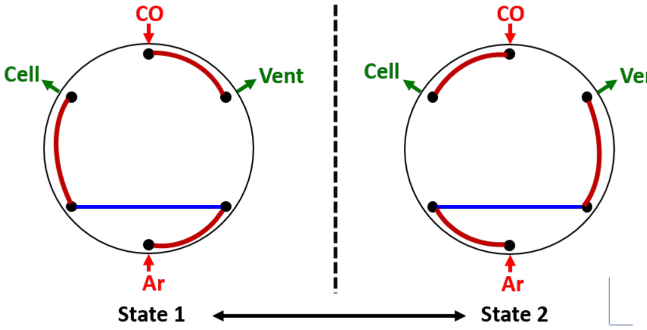

Figure S10. Operation switching valve of Valco Cheminert C25Z-3186UMH. In state 1, Ar is fed to the cell and CO is directed to the vent. In state 2, Ar is directed to the vent and CO is fed to the cell.

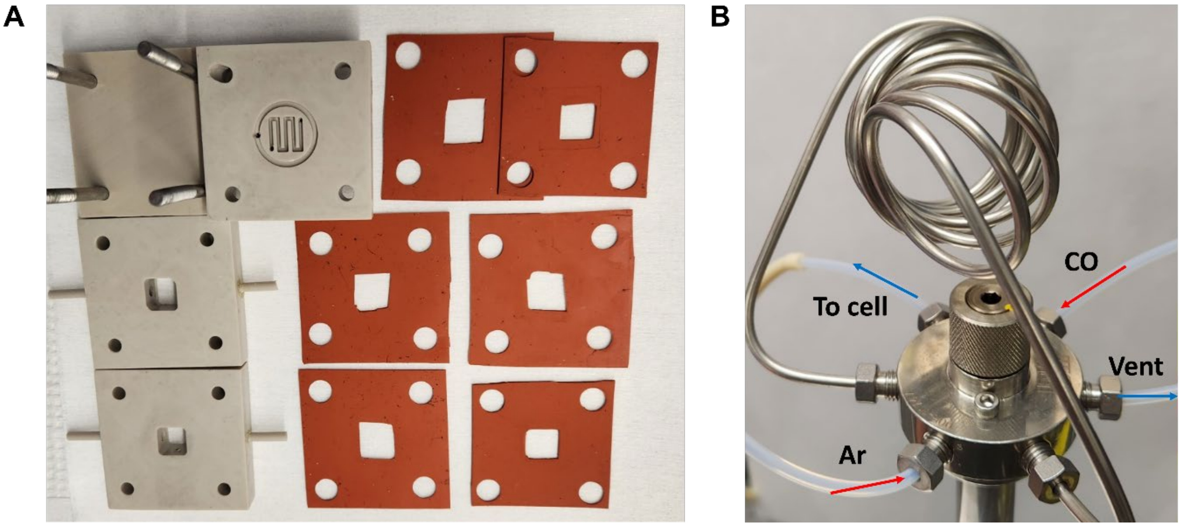

Figure S11. Photo images of GDE flow cell and gas switching valve. (A) Photo of gas diffusion electrode cell showing PEEK components (including the cathode flow field) and gaskets used for sealing. (B) Diagram of GC injection valve used to switch between Ar and CO flow. Two red arrows are the Ar and CO inlets to the valve. After passing through the valve, one is connected to the cell and the other goes to vent (blue arrows).

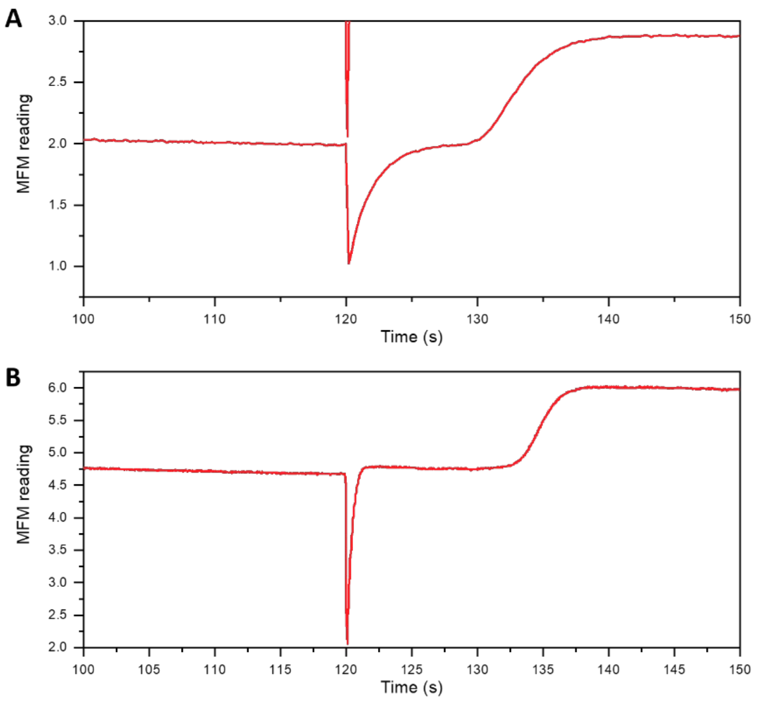

Figure S12. Importance of Pressure-Drop Mass Flow Meter for Improved Transient Resolution. MFM based on measurements of (A) thermal conductivity (B) pressure drop across an orifice are compared. With 5 sccm of CO gas flow and at -0.75V vs RHE applied potential in 1 M KOH electrolyte, CO gas flow was switched to 5 SCCM of Ar gas flow at 120 s. The time resolution of the MFM shown in (A) is not suitable for the present work.

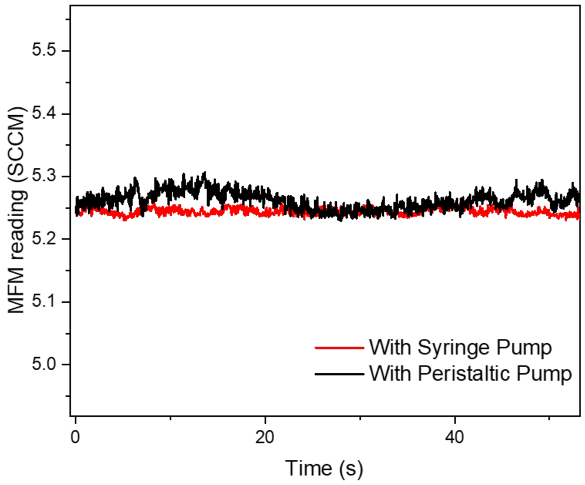

Figure S13. Reduction of noise in the MFM reading via use of syringe pump. As discussed above, the noise of MFM reading was effectively decreased when we used a syringe pump instead of a peristaltic pump. The syringe pump does not produce pressure pulses, in contrast to the peristaltic pump.

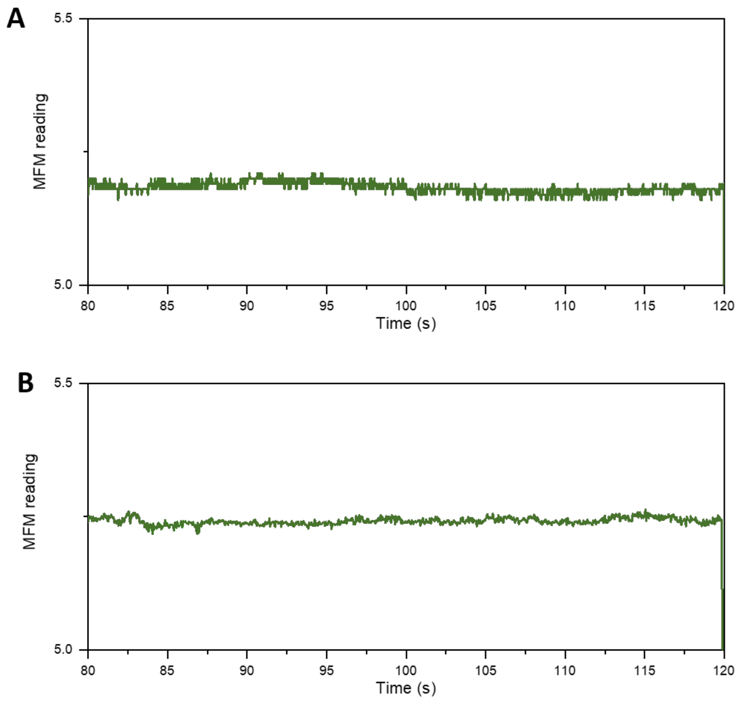

Figure S14. Mass flow measurement with improved digitization. MFM readout by (A) Alicat software with 30 Hz data rate and digital truncation and (B) analog output digitized at 20 Hz with high resolution (24-bit A/D).

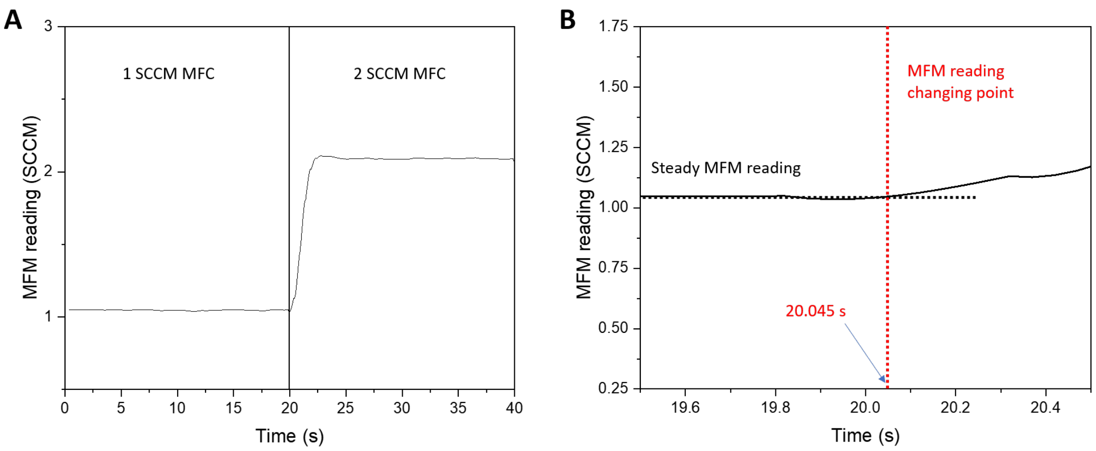

Figure S15. Instantaneous Response Times of Mass Flow Measurements. MFM was directly connected to MFC with tube length of 120.45 cm. A) Starting from 1 SCCM CO gas flow setting in MFC, gas flow was switched to 2 SCCM by MFC at 20.000 s. B) Mass flow measurements changed at 20.045 s. This small response time scale (0.045 s) is consistent with the manufacturers' specification of MFC change speed (30 ms) and MFM response time (10 ms).

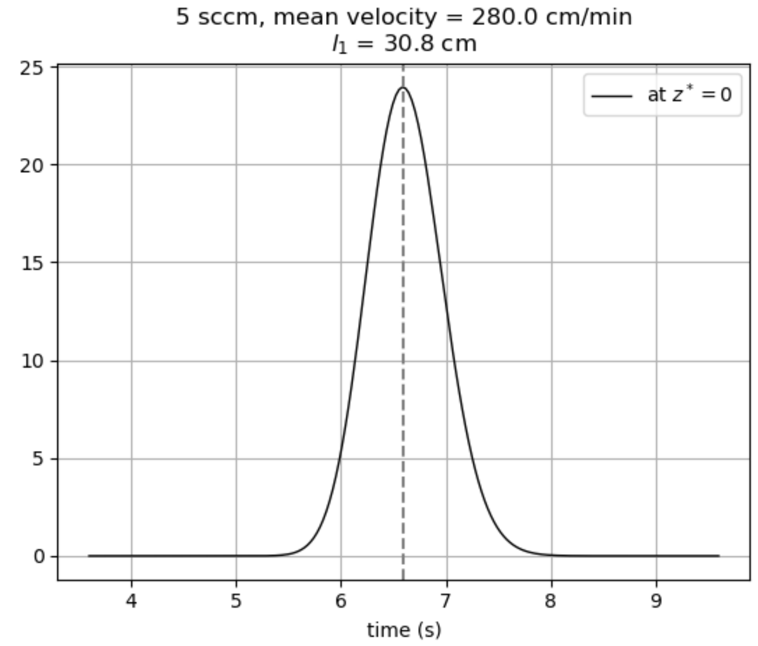

Figure S16. Simulation of Diffusional Broadening of Delta-Function Impulse of concentration. The temporal evolution of delta function was modeled using Taylor-Aris dispersion. The simulation was performed for typical conditions in this work: 5 sccm (mean velocity of 280 cm/minute at the temperature of the lab) gas flown in tube length L_1 = 30.8 cm corresponds to an arrival time t_1 = 6.60 s. The y-axis is concentration. While the maximum of the concentration signal arrives at 6.60 s, the diffusion along the length of the tubing means that the peak is not infinitely thin. The full width at half max of this profile is 0.82 s. Thus, the resolution of a transient in this experiment is expected to be very fast and approximately 0.8 s.

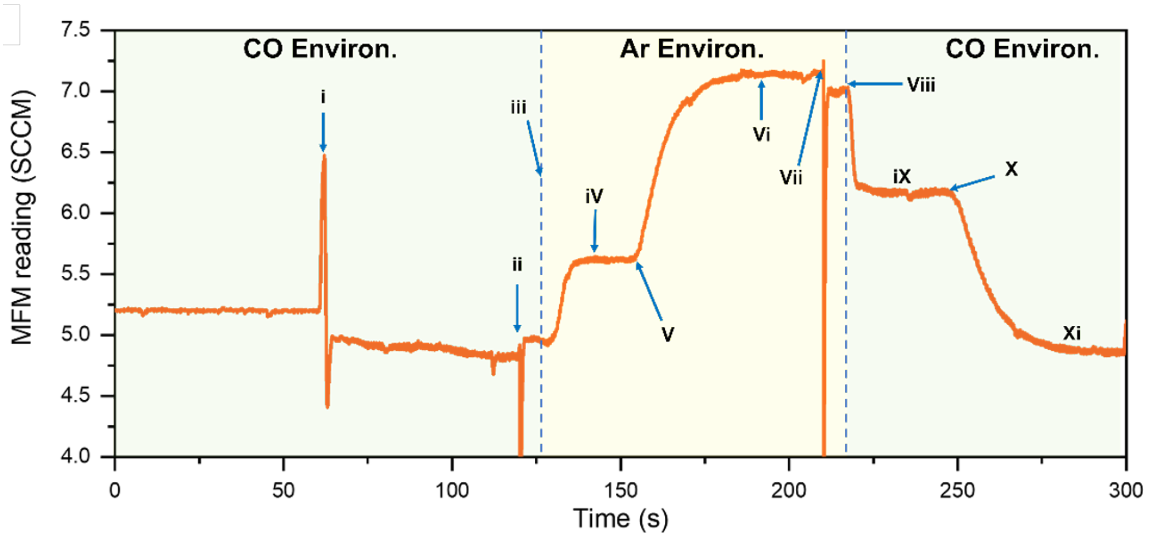

Figure S17. **Overall switching experiment from CO to Ar to CO.** The test was conducted in the system of OD Cu NPs at -1.52 V vs. SHE and L_1 = 30.83 cm, L_{cell} = 6.066 cm, L_2 = 111.68 cm and $\underline{u}$ = 4.60 cm/s. Each MFM response from i to Xi can be explained as follow: i) Pressure pulse from IR compensation, ii) pressure pulse from valve switching (120 s), iii) prediction (120 s + t_1 = 126.6 s) when Ar arrives to cell, iV) HER steady state before Ar arrives to MFM, V) viscosity response by Ar arrival to MFM, Vi) HER steady state with Ar reading, Vii) pressure pulse by valve switching (210 s), Viii) CO arrives to the entrance of the cell (210 s + t_1 = 216.6 s) and decrease of MFM reading by COR, iX) COR steady state before CO arrives to MFM, X) viscosity response by CO arrival to MFM, Xi) COR steady state with CO reading. Notably, after the arrival time for Ar to CO switch (point Viii), the MFM reading decreases due to COR nearly immediately (217.31 s, ~0.5 s later than point Viii), noting that 0.5 s corresponds well to the time required for the gas switch to reach the center of the cell. Therefore, the delay time for the Ar to CO switch (~0.5 s) is very small and is close to the relative time precision of the experiment.

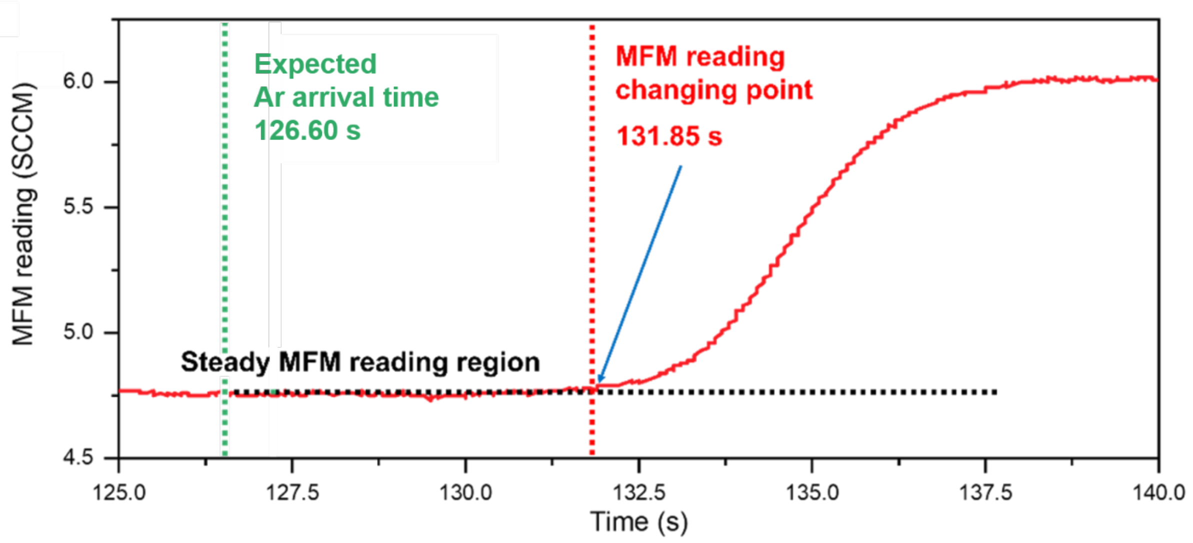

Figure S18. Determination of Delay Time. After a switch, the delay time is taken to be the time elapsed between feed gas arrival to the cell and the time that the mass flow signal switches 1% from the steady state value. In this example, at 120 s the gas feed switches from CO to Ar while the OD Cu catalyst is at -1.52 V vs. SHE in 1 M KOH electrolyte. The test was conducted in the system of L_1 = 30.83 cm, L_{cell} = 6.066 cm, L_2 = 111.68 cm and $\underline{u}$ = 4.60 cm/s. The expected arrival time for Ar to reach the cell is t_1 = 6.60 s. Thus, the delay time in this case is 131.85 s – 126.60 s = 5.25 s. The mass flow signal changes based on the increase in HER relative to C-C coupling. This procedure for determining delay time is also used for the gas switch of Ar to CO, using the same expected arrival time of t_1 = 6.60 s.

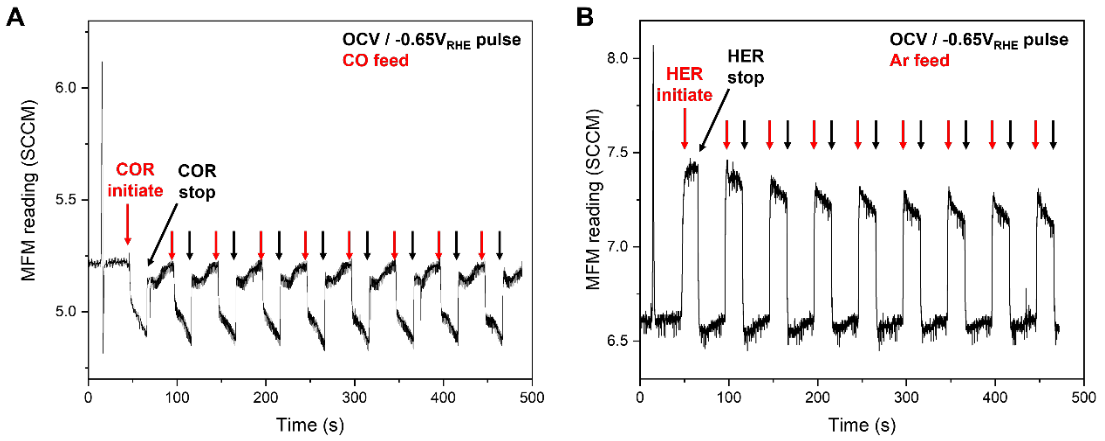

Figure S19. Characteristic mass flow measurements with repeatedly applied on/off potential. These experiments are conducted at 5 sccm of gas flow and using OD Cu NPs in 1 M KOH electrolyte. A) CO is fed to the catalyst system. Red arrows indicate that potential is

applied such that the CORR begins. HER is also present but not dominant, as noted by the mass flow reading being lower than the input feed. B) Ar is fed to the catalyst system. The MFM is calibrated to CO. Because Ar has higher viscosity than CO, the mass flow measurement will be higher for Ar than CO at the same flow rate. Ar with 5 sccm flow rate and no applied potential showed MFM reading of 6.62 sccm. Red arrows mean that potential is being applied such that hydrogen evolves from the electrolyte/catalyst (HER). Black arrows indicate the zero applied potential. Mass flow measurements of COR and HER were very fast, with rise and fall times of 0.14 s. Thus, the system shows time resolution for mass flow on the order of 150 ms.

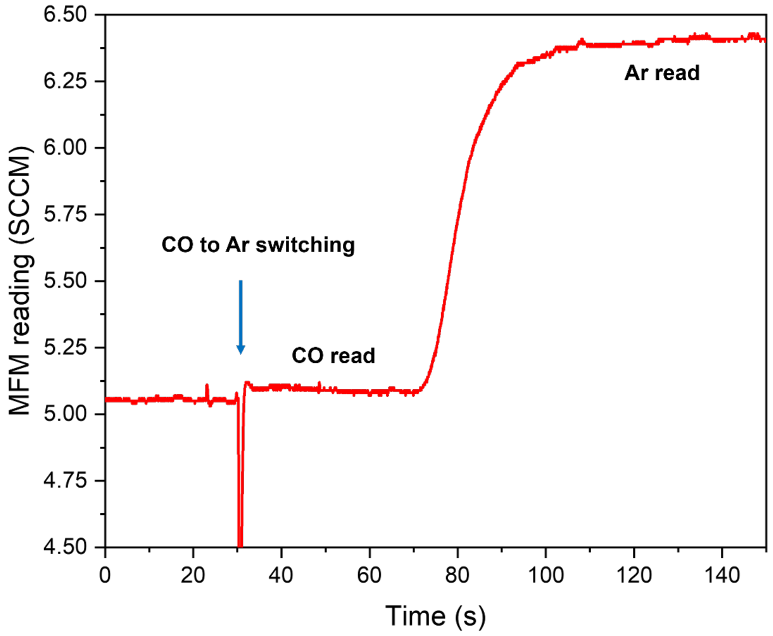

Figure S20. **Mass flow measurements without catalyst nor applied potential.** The test was conducted in the system of $L_1 = 30.83$ cm, $L_{cell} = 6.066$ cm, $L_2 = 143.42$ cm and $\underline{v} = 4.60$ cm/s. The MFCs for Ar and CO were both set to 5 sccm. At 30 s, the valve switches from CO to Ar. At 69.27 s, the MFM reads high because Ar is arriving to the MFM (Table S2). This demonstrates that the onset time (viscosity response time) of this tube length is around 40 seconds. This informed future experiments that any transient measurements of chemical surfaces must be done in those 40 seconds to be accurately described. For example, if the product composition were to change as CO is built up/scavenged from the surface (i.e. a shift between HER and C-C coupling), one could assess this within the 40 second window.

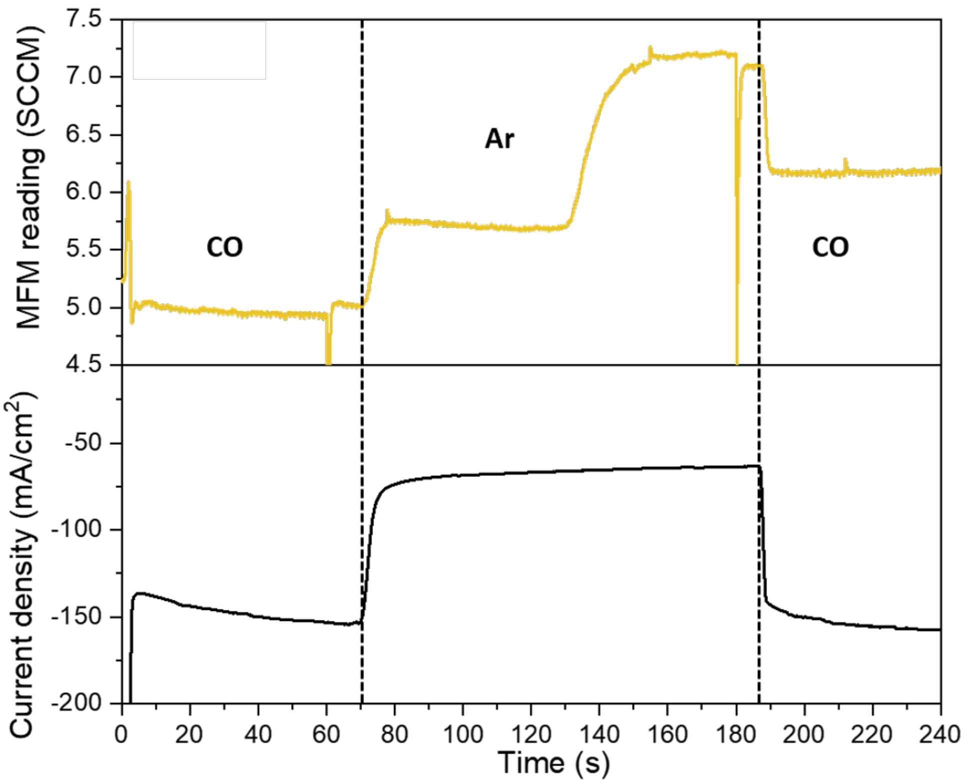

Figure S21. **Current density profile of overall switching experiment.** In this Figure, a potential (-1.62 V vs. SHE) is applied at 0 seconds to a catalyst system with CO feed. After switching to Ar at 60 s, current and mass flow start increasing at the same time (70.27 s) due to transition from COR to HER. After switching to CO again at 180 s, current and mass flow start decreasing at 187.31 s. Clearly, chemical transients observed via mass flow measurements occur nearly simultaneously with current change. These experiments utilized OD-Cu NPs (25 nm) in 1 M KOH electrolyte.

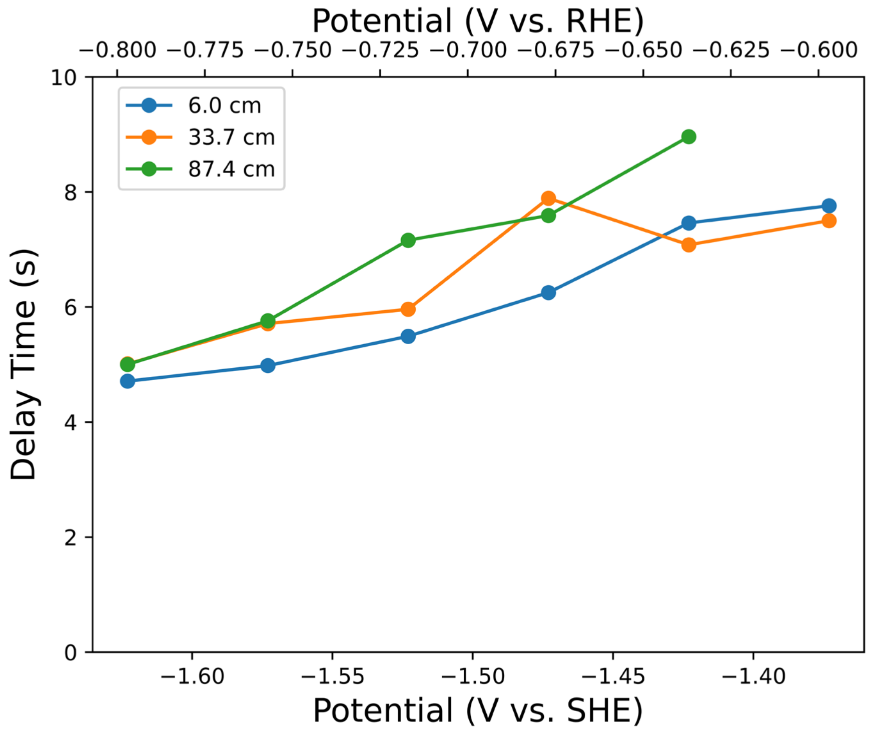

Figure S22. Control experiment of delay time with change of tube length L_1. L_1 tunes the interdiffusion of gases Ar and CO. Because the delay time always decreases with increasing negative potential, this demonstrates that tube length has negligible effect on delay time trends. For this experiment, the system was OD-Cu and Nafion in 1 M KOH electrolyte and 5 sccm Ar/CO.

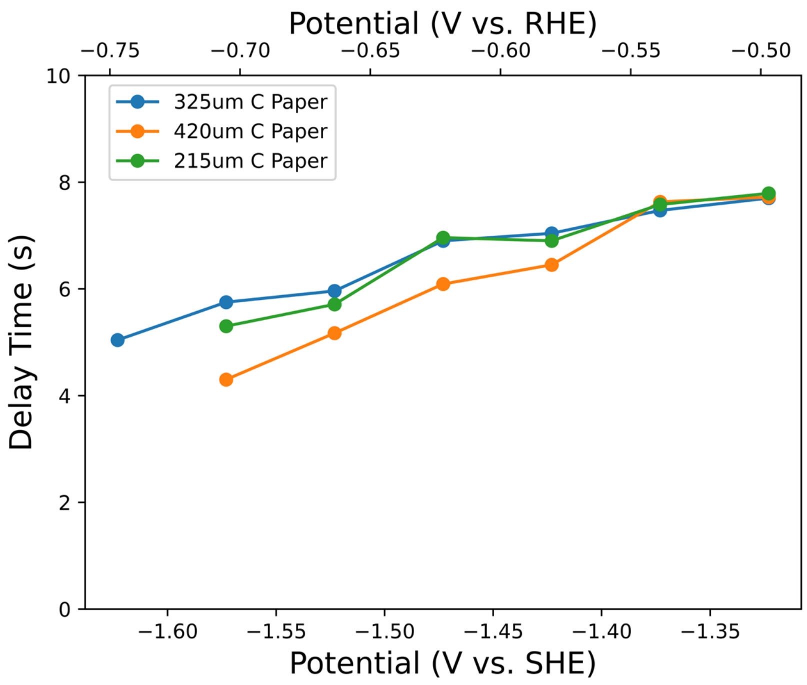

Figure S23. **Control experiment of delay time with change of C paper thickness.** The C paper thickness has negligible effect on delay time trends. For this experiment, the system was OD-Cu and Nafion in 1 M KOH electrolyte and 5 sccm Ar/CO.

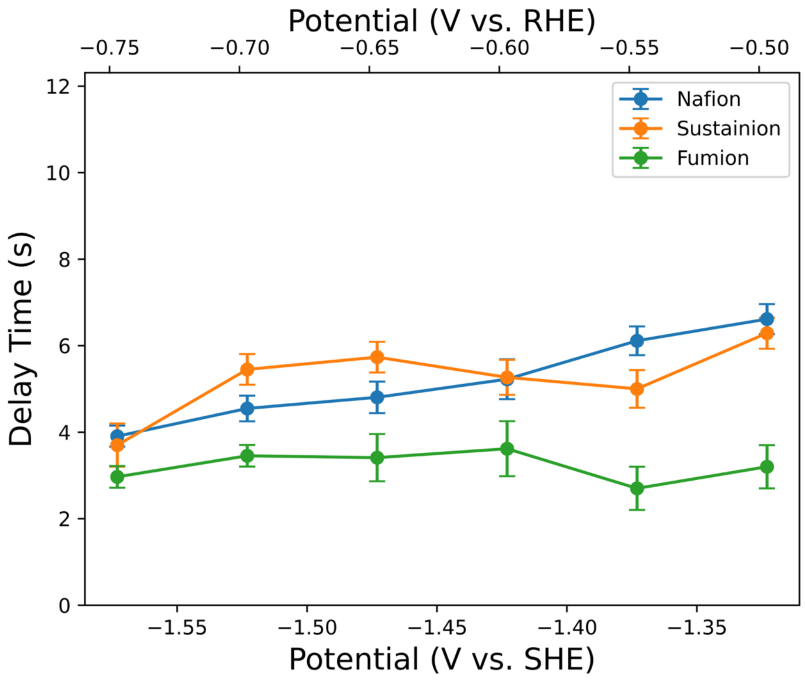

Figure S24. Control experiment of delay time with change of ionomer. This figure demonstrates that the ionomer has a relatively small effect on delay time. Catalyst was OD Cu dispersed in the respective ionomer solutions (same weight loading for Sustainion, Fumion, Nafion) in 1 M KOH electrolyte. Error bars are based on standard deviation.

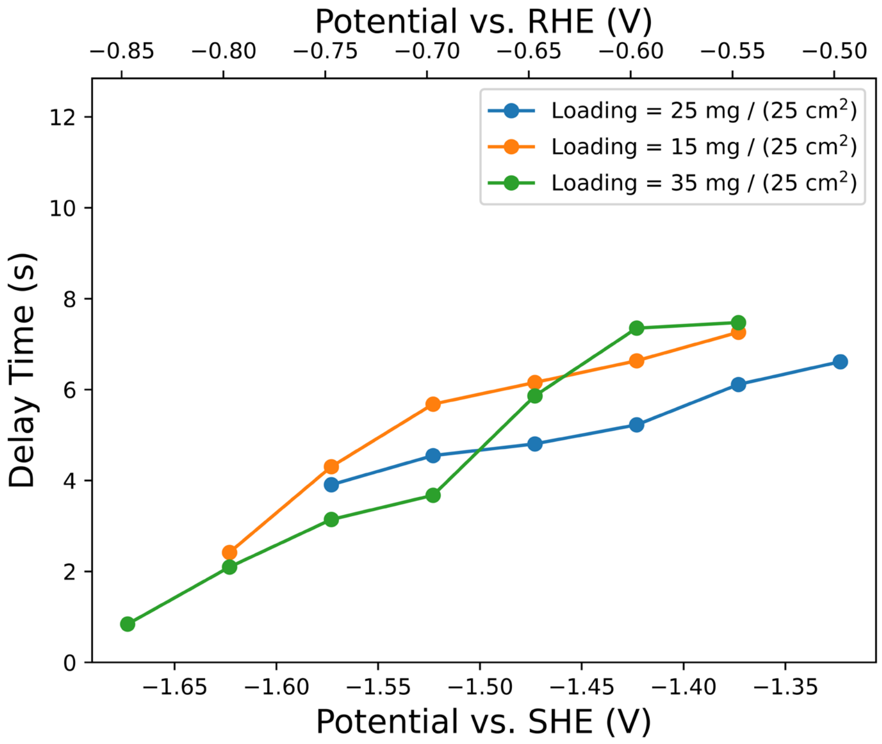

Figure S25. Control experiment of delay time with change of mass loading of OD Cu NP catalyst. From this graph, we see that the delay time has a similar relationship with overpotential as a function of catalyst loading. For these experiments, the system was OD-Cu and Nafion in 1 M KOH electrolyte and 5 sccm Ar/CO.

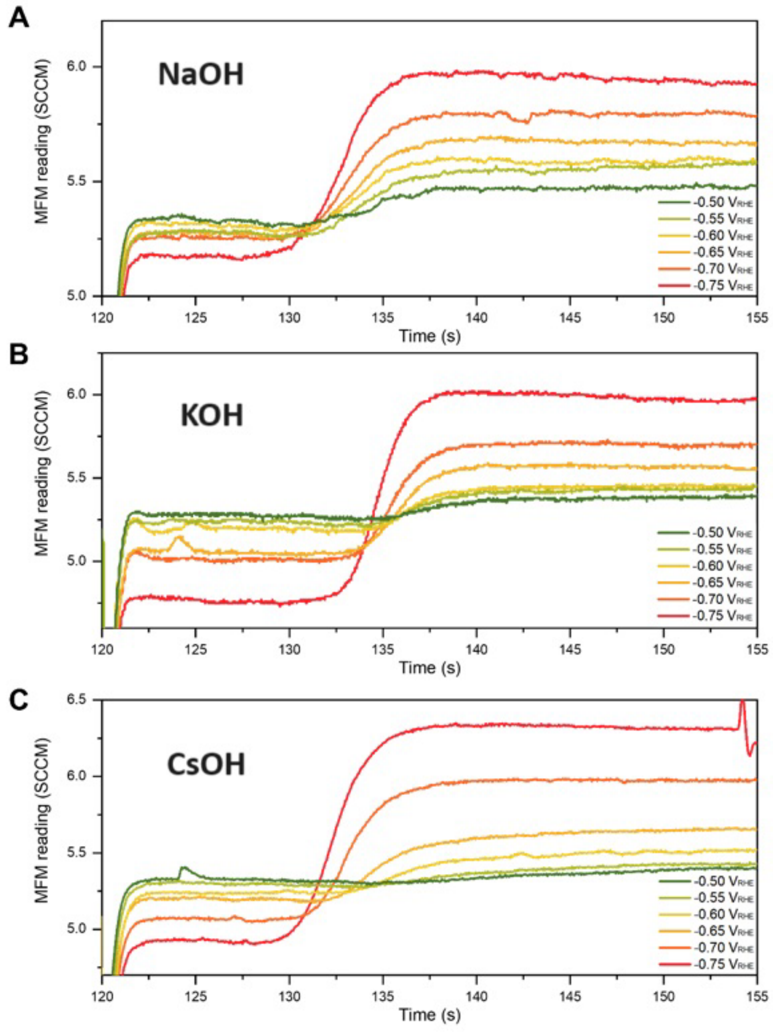

Figure S26. Effect of different cation size of electrolyte on mass flow measurement. MFM test was conducted by using three different 1 M hydroxide electrolytes with cation of (A) Na^+, (B) K^+, (C) Cs^+. System conditions are 25 nm OD-Cu and Nafion on C paper, 1 M (cation)OH, 1 mL/min electrolyte, 5 sccm gas flow rate, and partial CO pressure of 1 during the switch. Switch from CO to Ar occurs at 120 seconds, so the second transient is utilized to determine the delay time. Notably, the mass flow measurements as a function of applied potential are different which means product evolution changed during this time. Also, delay times vary as a function of potential and cation. These results are expounded upon in the main text (Figure 3).

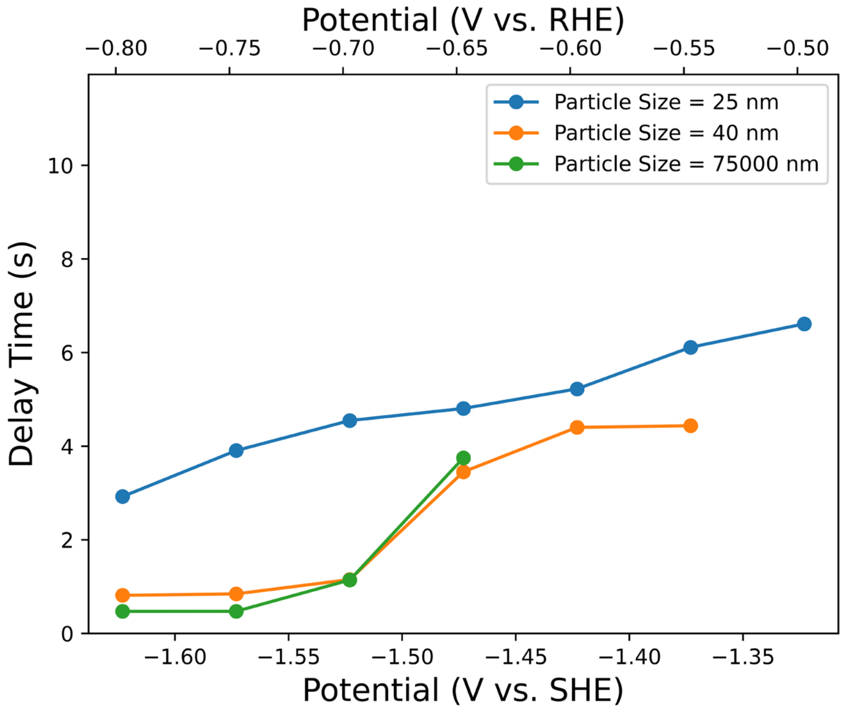

Figure S27. Effect of OD Cu original nanoparticle size on delay time. This demonstrates that the delay time is dependent on particle size. We hypothesize that this effect is due to a different distribution of catalyst sites and diffusion networks, but we do not fully explore this effect. More controlled experiments must be done to ascertain the role of nanoparticle size on delay time. For these experiments, the system was OD-Cu and Nafion in 1 M KOH electrolyte and 5 sccm Ar/CO.

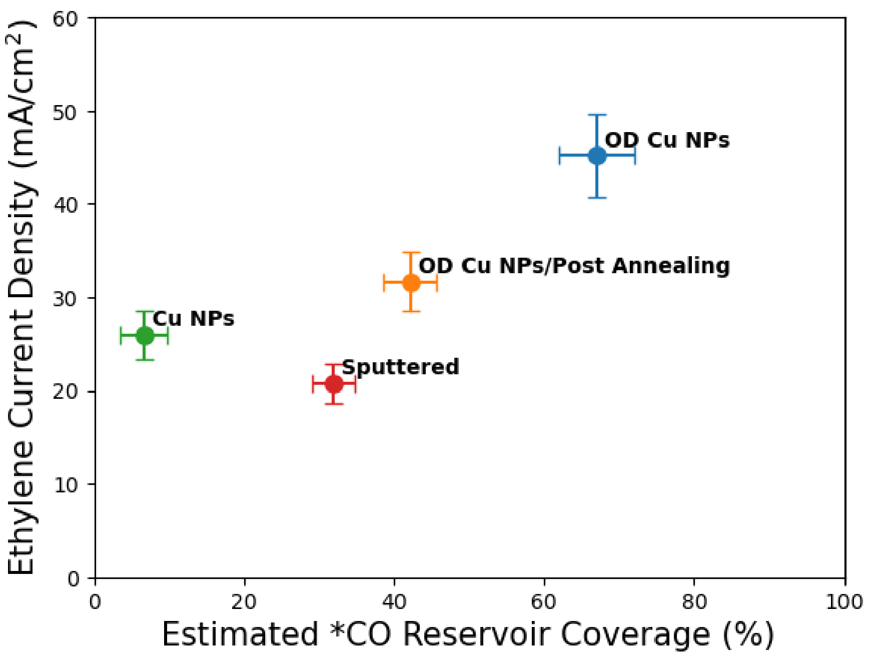

Figure S28. Partial current density to ethylene as a function of *CO reservoir coverage. This graph is similar to **Figure 5** in the main text, except current density is used as the activity metric. Datapoints are from various Cu preparations in 1 M KOH and 1 bar CO at an applied voltage of -1.47 V vs SHE.

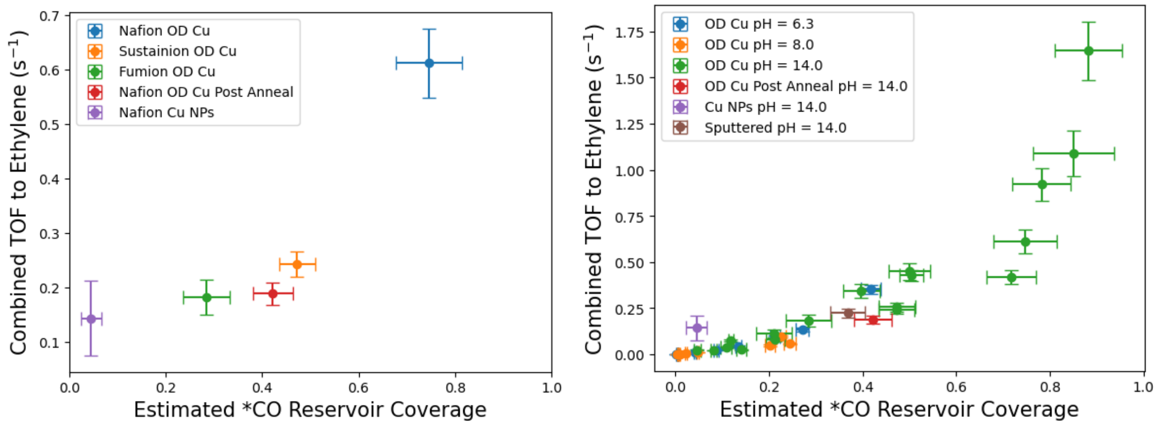

Figure S29. Evidence that *CO reservoir coverage increases combined turnover frequency to ethylene. Left: Plots of various Cu nanoparticle preparations including different ionomers and as-purchased, oxide-derived, and post-annealed. This data comes from standard cell conditions of -1.47 V vs SHE in 1M KOH electrolyte and 1 bar Ar/CO during the switch. Right: Combined TOF to ethylene as a function of all preparation conditions screened in this work, including data from different applied potentials, different ionomers, different pHs, and different preparations. This demonstrates that general trends shown in the main text may hold true regardless of applied potential. Notably, most data in this work is focused on OD Cu, so the near-exponential functional relationship may not be true for other synthetic methods.

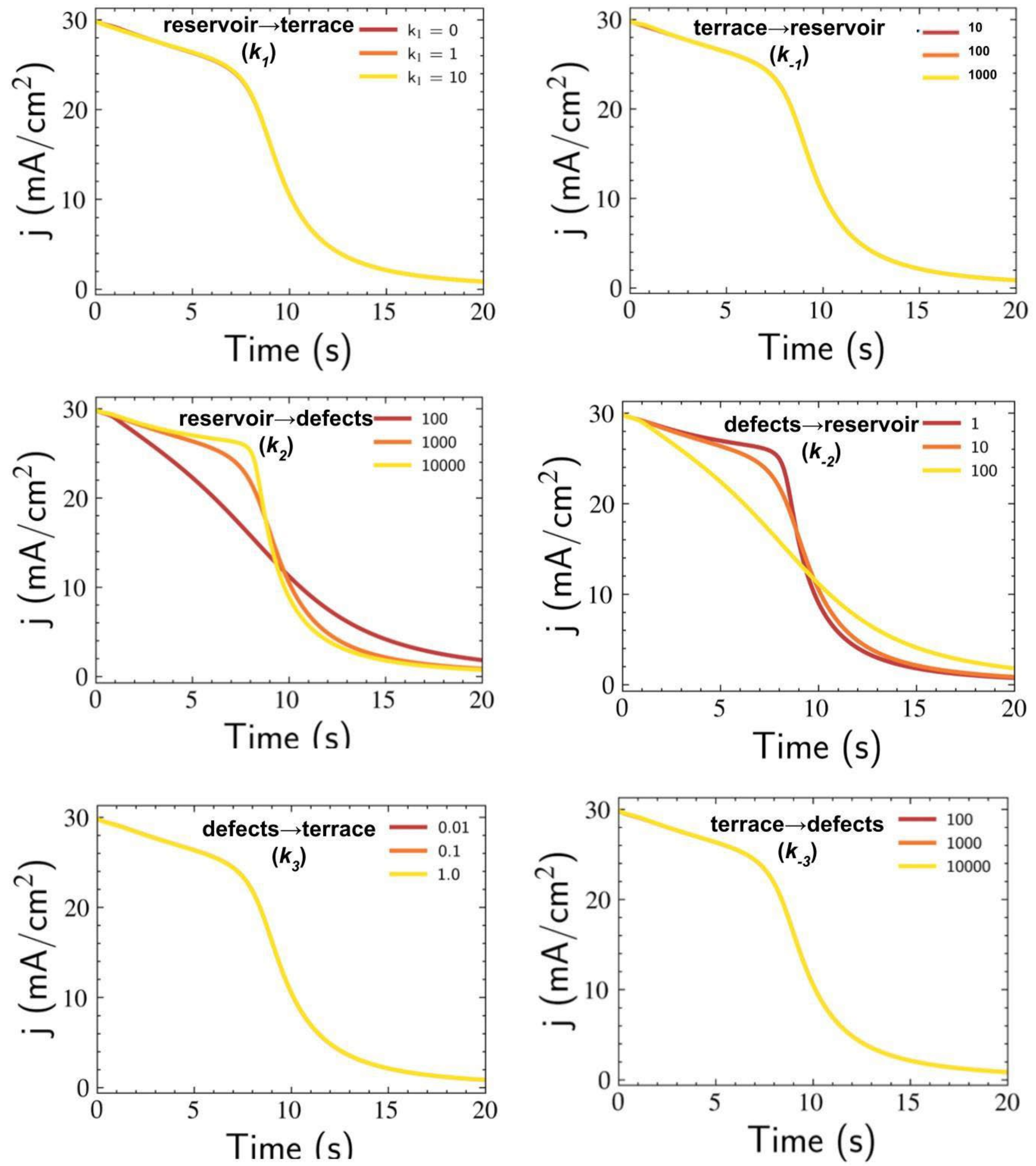

Figure S30. Effect of diffusion constants on current density and delay time. There is no effect on steady state current density ($j|_{\text{t = 0 s}}$). Only the supply of CO between reservoirs and defects lead to a noticeable change in delay time phenomena.

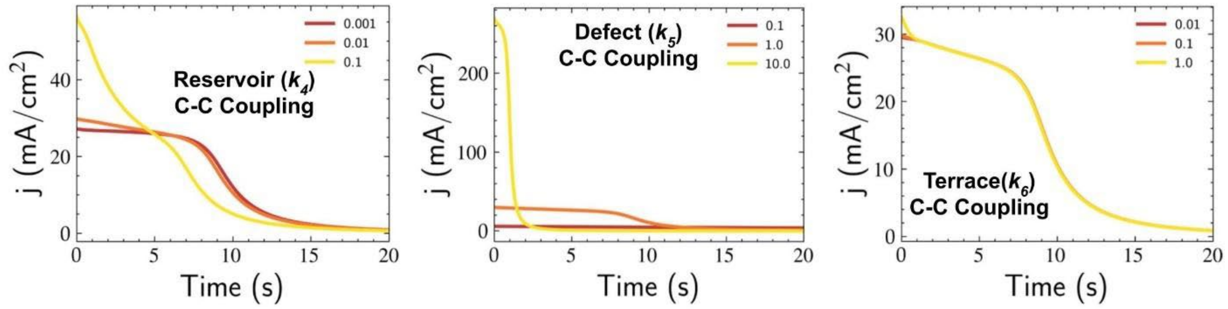

Figure S31. Effect of C-C coupling rate constants on current density and delay time. Increasing C-C coupling rate for any site increases steady state current density ($j|_{t\,=\,0\,s}$). The reservoir must be relatively inactive for long delay times to be observed. Also, increasing activity of defect sites increases CO consumption rates and lowers delay time.

Supplemental references

(1) Verdaguer-Casadevall, A.; Li, C. W.; Johansson, T. P.; Scott, S. B.; McKeown, J. T.; Kumar, M.; Stephens, I. E. L.; Kanan, M. W.; Chorkendorff, I. Probing the Active Surface Sites for CO Reduction on Oxide-Derived Copper Electrocatalysts. *J Am Chem Soc* **2015**, *137* (31), 9808–9811. https://doi.org/10.1021/jacs.5b06227.

(2) Prabhakar, R. R.; Lemerle, R.; Barecka, M.; Kim, M.; Seo, S.; Dayi, E. N.; Dei Tos, I.; Ager, J. W. TaOx Electron Transport Layers for CO2 Reduction Si Photocathodes. *J Mater Chem A Mater* **2023**, *11* (25), 13588–13599. https://doi.org/10.1039/d3ta01028g.

(3) Chatterjee, T.; Boutin, E.; Robert, M. *Manifesto for the Routine Use of NMR for the Liquid Product Analysis of Aqueous CO 2 Reduction: From Comprehensive Chemical Shift Data to Formaldehyde Quantification in Water*; 2020.

(4) Singh, M. R.; Kwon, Y.; Lum, Y.; Ager, J. W.; Bell, A. T. Hydrolysis of Electrolyte Cations Enhances the Electrochemical Reduction of CO2 over Ag and Cu. *J Am Chem Soc* **2016**, *138* (39), 13006–13012. https://doi.org/10.1021/jacs.6b07612.

(5) Hammer, B.; Hansen, L. B.; Nørskov, J. K. Improved Adsorption Energetics within Density-Functional Theory Using Revised Perdew-Burke-Ernzerhof Functionals. *Phys Rev B Condens Matter Mater Phys* **1999**, *59* (11), 7413–7421. https://doi.org/10.1103/PhysRevB.59.7413.

(6) Kresse, G.; Furthmiiller B ', J. *Efficiency of Ab-Initio Total Energy Calculations for Metals and Semiconductors Using a Plane-Wave Basis Set*; 1996; Vol. 6.

(7) Ong, S. P.; Richards, W. D.; Jain, A.; Hautier, G.; Kocher, M.; Cholia, S.; Gunter, D.; Chevrier, V. L.; Persson, K. A.; Ceder, G. Python Materials Genomics (Pymatgen): A Robust, Open-Source Python Library for Materials Analysis. *Comput Mater Sci* **2013**, *68*, 314–319. https://doi.org/10.1016/j.commatsci.2012.10.028.

(8) Tran, R.; Xu, Z.; Radhakrishnan, B.; Winston, D.; Sun, W.; Persson, K. A.; Ong, S. P. Surface Energies of Elemental Crystals. *Sci Data* **2016**, *3* (1), 160080. https://doi.org/10.1038/sdata.2016.80.

(9) Mathew, K.; Sundararaman, R.; Letchworth-Weaver, K.; Arias, T. A.; Hennig, R. G. Implicit Solvation Model for Density-Functional Study of Nanocrystal Surfaces and

Reaction Pathways. *Journal of Chemical Physics* **2014**, *140* (8). https://doi.org/10.1063/1.4865107.

(10) Mathew, K.; Kolluru, V. S. C.; Mula, S.; Steinmann, S. N.; Hennig, R. G. Implicit Self-Consistent Electrolyte Model in Plane-Wave Density-Functional Theory. *Journal of Chemical Physics* **2019**, *151* (23). https://doi.org/10.1063/1.5132354.

(11) Duan, Z.; Xiao, P. Simulation of Potential-Dependent Activation Energies in Electrocatalysis: Mechanism of O-O Bond Formation on RuO2. *Journal of Physical Chemistry C* **2021**, *125* (28), 15243–15250. https://doi.org/10.1021/acs.jpcc.1c02998.

(12) Clark, I. O.; Fripp, A. L.; Jesser, W. A. MOCVD Manifold Switching Effects on Growth and Characterization. *J Cryst Growth* **1991**, *109* (1–4), 246–251. https://doi.org/10.1016/0022-0248(91)90186-9.

(13) *PyVisa. https://pyvisa.readthedocs.io/en/latest/.*

(14) *NI-DAQmx Python API. https://nidaqmx-python.readthedocs.io/en/latest/.*

(15) *Alicat Gas SelectTM 5.0 Preloaded Gases and Properties https://documents.alicat.com/specifications/Alicat_Preloaded-Gases-and-Properties_Rev0.pdf (accessed Apr 23, 2023).*

(16) Shannon, S. L.; Goodwin, J. G. *Characterization of Catalytic Surfaces by Isotopic-Transient Kinetics during Steady-State Reaction*; 1995; Vol. 95. https://pubs.acs.org/sharingguidelines.

(17) Berger, R. J.; Kapteijn, F.; Moulijn, J. A.; Marin, G. B.; De Wilde, J.; Olea, M.; Chen, D.; Holmen, A.; Lietti, L.; Tronconi, E.; Schuurman, Y. Dynamic Methods for Catalytic Kinetics. *Applied Catalysis A: General*. June 30, 2008, pp 3–28. https://doi.org/10.1016/j.apcata.2008.03.020.

(18) Dryer, F. L.; Haas, F. M.; Santner, J.; Farouk, T. I.; Chaos, M. Interpreting Chemical Kinetics from Complex Reaction-Advection-Diffusion Systems: Modeling of Flow Reactors and Related Experiments. *Progress in Energy and Combustion Science*. Elsevier Ltd 2014, pp 19–39. https://doi.org/10.1016/j.pecs.2014.04.002.

(19) Fuller, E. N.; Schettler, P. D.; Giddings, J. Calvin. NEW METHOD FOR PREDICTION OF BINARY GAS-PHASE DIFFUSION COEFFICIENTS. *Ind Eng Chem* **1966**, *58* (5), 18–27. https://doi.org/10.1021/ie50677a007.

(20) Taylor, G. Conditions under Which Dispersion of a Solute in a Stream of Solvent Can Be Used to Measure Molecular Diffusion. *Proc R Soc Lond A Math Phys Sci* **1954**, *225* (1163), 473–477. https://doi.org/10.1098/rspa.1954.0216.

(21) Aris, R. On the Dispersion of a Solute in a Fluid Flowing through a Tube. *Proc R Soc Lond A Math Phys Sci* **1956**, *235* (1200), 67–77. https://doi.org/10.1098/rspa.1956.0065.

(22) Choi, W.; Park, S.; Jung, W.; Won, D. H.; Na, J.; Hwang, Y. J. Origin of Hydrogen Incorporated into Ethylene during Electrochemical CO2 Reduction in Membrane Electrode Assembly. *ACS Energy Lett* **2022**, *7* (3), 939–945. https://doi.org/10.1021/acsenergylett.1c02658.

(23) Amini, K.; Sadeghi, A.; Pritzker, M.; Gostick, J. Porous Electrodes in Redox Flow Batteries. In *Encyclopedia of Energy Storage: Volume 1-4*; Elsevier, 2022; Vol. 1–4, pp 466–479. https://doi.org/10.1016/B978-0-12-819723-3.00064-0.

(24) Tomadakis, M. M.; Robertson, T. J. Viscous Permeability of Random Fiber Structures: Comparison of Electrical and Diffusional Estimates with Experimental and Analytical Results. *J Compos Mater* **2005**, *39* (2), 163–188. https://doi.org/10.1177/0021998305046438.

(25) Li, J.; Maresi, I.; Lum, Y.; Ager, J. W. Effects of Surface Diffusion in Electrocatalytic CO2reduction on Cu Revealed by Kinetic Monte Carlo Simulations. *Journal of Chemical Physics* **2021**, *155* (16). https://doi.org/10.1063/5.0068517.
